# Unveiling the gas-sensing potential of methylene-bridged [6]cycloparaphenylene nanobelt: a DFT perspective

**DOI:** 10.1038/s41598-025-28684-1

**Published:** 2025-11-23

**Authors:** Rima Heider Al Omari, Shelesh Krishna Saraswat, Munthar Kadhim Abosaoda, M. M. Rekha, Subhashree Ray, Kattela Chennakesavulu, Renu Sharma, Aditya Kashyap

**Affiliations:** 1 Department of Medical Laboratory Sciences, Faculty of Allied Medical Sciences, Hourani Center for Applied Scientific Research, Al-Ahliyya Amman University, Amman, Jordan; 2 Department of Electronics and Communication Engineering, GLA University, Mathura, 281406 India; 3 College of Pharmacy, The Islamic University, Najaf, Iraq; 4 College of Pharmacy, The Islamic University of Al Diwaniyah, Al Diwaniyah, Iraq; 5 Department of Chemistry and Biochemistry, School of Sciences, JAIN (Deemed-to- be University), Bangalore, Karnataka India; 6 Department of Biochemistry, IMS and SUM Hospital, Siksha ’O’ Anusandhan (Deemed to be University), Bhubaneswar, Odisha 751003 India; 7 Department of Chemistry, Sathyabama Institute of Science and Technology, Chennai, Tamil Nadu India; 8 Department of Chemistry, University Institute of Sciences, Chandigarh University, Mohali, Punjab India; 9 Centre for Research Impact and Outcome, Chitkara University Institute of Engineering and Technology, Chitkara University, Rajpura, Punjab 140401 India

**Keywords:** Methylene-bridged [6]cycloparaphenylene, Gas-sensing, DFT, Nanobelt, Chemistry, Materials science, Physics

## Abstract

The rapid expansion of industrialization has led to the release of numerous toxic gases into the atmosphere, posing significant environmental and health risks even at low concentrations. This has created an urgent need for novel carbon-based nanostructures capable of detecting gases selectively and efficiently. In this study, we systematically investigate the adsorption behavior and sensing performance of the methylene-bridged [6]cycloparaphenylene ([6]MCPP) nanobelt for detecting hazardous gases, including hydrogen sulfide (H₂S), carbon monoxide (CO), ammonia (NH₃), ozone (O₃), sulfur dioxide (SO₂), and nitrogen dioxide (NO₂), using density functional theory (DFT)-based electronic structure analysis. The [6]MCPP nanobelt demonstrated excellent structural robustness, with a cohesive energy of − 301.22 kcal/mol, confirming its intrinsic stability for gas-sensing applications. Among the gases examined, NO₂ exhibited the most pronounced interaction-induced electronic modulation, reflected by a 67.17% reduction in the HOMO–LUMO energy gap (from 2.62 to 0.84 eV) and a sharp rise in electrical conductivity (up to 239 S/m). Its rapid recovery time of 978 µs (9.78 × 10⁻⁴ s) at 298 K suggests both high sensitivity and excellent reusability. Although SO₂ showed the strongest adsorption energy (-20.8 kcal/mol), the moderate binding strength of -12.33 kcal/mol for NO₂, combined with its large band gap variation and short recovery time, highlights an optimal balance between selectivity and reversibility. Density of states (DOS) analysis revealed significant redistribution of electronic states near the Fermi level, confirming enhanced charge transfer upon gas adsorption. Additionally, quantum theory of atoms in molecules (QTAIM), electron localization function (ELF), and non-covalent interaction (NCI) analyses identified van der Waals forces as the dominant stabilizing interactions, with notably strong attractive regions for [6]MCPP@O₃, [6]MCPP@SO₂, and [6]MCPP@NO₂ complexes. These findings not only establish [6]MCPP as a stable and reusable nanobelt sensor but also reveal its superior selectivity and sensitivity toward NO₂, positioning it as a next-generation candidate for efficient environmental monitoring applications.

**Supplementary Information:**

The online version contains supplementary material available at 10.1038/s41598-025-28684-1.

## Introduction

The rapid expansion of industrial activities and the continuous reliance on fossil fuels have resulted in the release of toxic gases into the atmosphere, posing severe threats to public health and environmental safety. The World Health Organization (WHO) identifies air pollution, driven by hazardous gases such as hydrogen sulfide (H₂S), carbon monoxide (CO), ammonia (NH₃), ozone (O₃), sulfur dioxide (SO₂), and nitrogen dioxide (NO₂), as a leading contributor to chronic respiratory diseases, including asthma, cystic fibrosis, bronchitis, and lung cancer^1–8^. Even at low concentrations, these gases exhibit significant toxicity. Hydrogen sulfide (H₂S), a flammable and corrosive gas with a characteristic pungent odor, is primarily produced in oil refineries and becomes poisonous above 250 ppm^9,10^.

Carbon monoxide (CO), an odorless and colorless product of incomplete combustion, poses a serious threat when its concentration exceeds 35 ppm^11^. Ammonia (NH₃), a colorless gas with a sharp odor, becomes harmful to human health when atmospheric concentrations surpass 25 ppm^12^.

According to the WHO, exposure to ozone (O₃) levels above 75 ppb can trigger lung-related illnesses^13^. Sulfur dioxide (SO₂), commonly generated during fossil fuel combustion and metal extraction processes, leads to respiratory complications after prolonged exposure to concentrations above 20 ppm^14^. Similarly, nitrogen dioxide (NO₂), a highly toxic oxidizing gas predominantly emitted from vehicle exhaust, adversely affects the respiratory system even at levels as low as 1 ppm^15,16^. These facts underscore the urgent need for the development of advanced gas-sensing materials capable of high sensitivity, selectivity, and strong adsorption efficiency toward these hazardous gases.

**Fig. 1:**
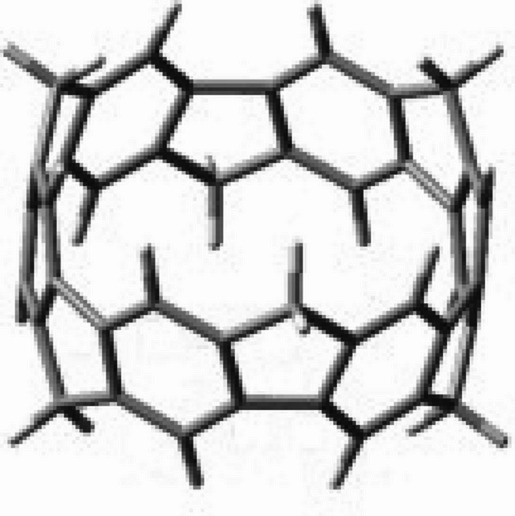
Molecular structure of [6]cycloparaphenylene ([6]MCPP).

Among emerging candidates, [n]Cycloparaphenylenes ([n]CPPs), a class of cyclic nanocarbons composed of benzene rings linked at their para-positions, have gained prominence as model systems in supramolecular chemistry^17–19^. Since the pioneering synthesis of CPPs by Jasti et al. (2008), numerous derivatives have been developed, revealing unique electronic, optical, and structural characteristics^20–22^. One notable advancement was reported by Li et al., who synthesized a methylene-bridged [6]cycloparaphenylene ([6]MCPP), introducing a nonalternant aromatic system^23^. As shown in Fig. 1, this structure incorporates methylene bridges that interconnect neighboring paraphenylene units. Compared with its parent compound [6]CPP, the methylene bridges in [6]MCPP enforce a coplanar orientation between adjacent phenylene rings, thereby enhancing π-conjugation and reducing the energy gap to 2.66 eV^24^. Structurally, the [6]MCPP nanobelt can be considered a fundamental segment of more complex carbon nanostructures, including Haeckelite nanotubes (composed of pentagon–hexagon–heptagon motifs) and fullerenes such as Ih-C₈₀^23^. Moreover, its intrinsic cavity allows small molecule encapsulation, making it an excellent platform for optoelectronic and gas-sensing applications.

Previous density functional theory (DFT)-based investigations have extensively demonstrated the capability of nanomaterials to engage in both physisorption and chemisorption with diverse gas molecules^25–29^. For example, Patel et al.^30^ examined the gas-sensing behavior of C₁₂, B₆N₆, and Al₆N₆ nanoclusters toward CO, NO, and NH₃ molecules. Their findings revealed that the interaction mechanism strongly depends on the substrate type: the C₁₂ nanocluster exhibited chemisorption with CO and NO, whereas its interaction with NH₃ was physisorptive. In contrast, the B₆N₆ nanocluster displayed chemisorption toward NH₃ and physisorption toward CO and NO, while the Al₆N₆ nanocluster exhibited only weak physisorption with all three gas species.

To further elucidate the structure–property correlation, Sainda et al.^31^ investigated the Mg₉O₉ nanoring as a potential adsorbent and sensor for CO, NO, and NH₃ gases. Their results indicated strong adsorption of NO and NH₃, characterized by high adsorption energies and short binding distances, whereas the interaction with CO remained weak and primarily physisorptive. Additionally, Rhrissi et al.^32^ demonstrated that adsorption properties can be effectively tuned through strain engineering, showing that the adsorption energy of NO₂ on BC₂N nanosheets increased notably from −  0.43 eV to − 0.68 eV under applied mechanical strain, accompanied by enhanced charge transfer between the gas molecule and substrate.

In addition, Abbasi et al.^33^ investigated the adsorption and electronic properties of Ag_₂_O-modified BP/BSe van der Waals heterostructures, finding that NO₂ exhibited the highest adsorption energy (-2.64 eV) and strong chemisorption, indicating excellent sensing capability. Abbasi and Sardroodi^34^ studied SO_x_ adsorption on N-doped ZnO nanoparticles, revealing higher adsorption energies and multiple contact points compared to pristine ZnO^35^, with PDOS analysis confirming covalent interactions between the adsorbate and nanoparticle. Furthermore, CO and NO adsorption on Al-, P-, and Si-doped MoS₂ monolayers demonstrated stronger binding and shorter adsorption distances on doped systems, with PDOS confirming chemical bond formation^36^. Another study showed that TiO₂/Stanene heterostructures were found to chemically adsorb NO₂ and O₃, indicating covalent interactions^37^.

The efficacy of nanocages for gas capture has been demonstrated by Rani et al.^38^, who reported that the CC₁ nanocage could efficiently trap SOCl₂ and SF₄, but exhibited only weak adsorption toward H₂S. The selectivity of specific gases was highlighted by Sattar et al.^39^, who identified kekulene as a promising selective adsorbent for SO₂, with adsorption energies ranging from −  2.81 to − 4.88 eV. Conversely, Lal et al.^40^ suggested that the C₉N₄ surface interacts predominantly via physisorption with CO, NO, CO₂, HF, and COCl₂, with the highest adsorption energy being a modest − 0.95 eV for COCl₂.

Given that the gas-sensing potential of pristine [6]MCPP remains unexplored, the present study investigates its applicability as a sensor for detecting toxic gases, including hydrogen sulfide (H₂S), carbon monoxide (CO), ammonia (NH₃), ozone (O₃), sulfur dioxide (SO₂), and nitrogen dioxide (NO₂), using density functional theory (DFT) calculations. We systematically examine the changes in the structural, electronic, optical, and thermodynamic properties of [6]MCPP upon interaction with these gases. This analysis employs frontier molecular orbital (FMO) theory, UV–Vis spectroscopy, charge decomposition analysis (CDA), and electron density difference (EDD) maps. Furthermore, the nature of intermolecular interactions is elucidated using the quantum theory of atoms in molecules (QTAIM), electron localization function (ELF), and non-covalent interaction (NCI) analysis.

## Computational methods

Structural optimization was performed using the Gaussian 16 program package^41^ at the B3LYP/6-311 + + G(d, p) level of theory. The empirical dispersion correction (D3) developed by Grimme was explicitly included to account for van der Waals (vdW) interactions^42^. All calculations were performed without imposing any constraints on the potential energy surface, and frequency analysis showed no negative frequencies, indicating that the optimized structures correspond to true minima. The optimized structures and molecular orbitals were visualized using GaussView 6.0 software^43^.

The stability of the free [6]MCPP nanobelt was evaluated by calculating its cohesive energy (E_coh_). The stability of the resulting adsorption complexes ([6]MCPP@gas) was assessed based on their interaction energy (E_int_). These energies were calculated using the following equations:

1 $${E_{Coh}}={\mathrm{~}}\frac{1}{N}{\mathrm{~}}\left[ {{E_{[6]MCPP}}{\mathrm{~}} - \mathop \sum \limits_{y} {n_y}{E_y}} \right]$$

2 $${E_{int}}={E_{\left[ 6 \right]MCPP@gas~}} - \left( {{E_{MCPP}}+{E_{gas}}} \right)$$

where N, n_y_, and E_y_ are the total number of atoms in the [6]MCPP, the number of y species (y = H or C atoms), and the energy of isolated y species, respectively. E_[6]MCPP@gas_, E_MCPP_, and E_gas_ represent the energy of the gas-adsorbed complexes, the MCPP, and isolated gas molecules, respectively. The interaction energies were corrected by using counterpoise (CP) method^44^ as given in following equation:

3 $${E_{cp,int}}=~{E_{int}}~+~BSSE$$

where E_cp., int_ and BSSE are the counterpoise corrected interaction energy and basis set superposition error, respectively.

To evaluate the extent of structural deformation of the nanobelt upon gas adsorption, the deformation energy (E_def_) of each [6]MCPP@gas complex was determined according to the following equation^45^:

4 $${E_{def}}={E_{\left[ 6 \right]MCCP}} - {E_{\left[ 6 \right]MCCP}}\;in\;complex$$

Key thermodynamic parameters, including the change in enthalpy (ΔH), Gibbs free energy (ΔG), and entropy (ΔS), were calculated using the following equations:

5 $$\Delta G{\text{ }}={G_{\left[ 6 \right]MCPP@gas~}} - \left( {{G_{MCPP}}+{G_{gas}}} \right)$$

6 $$\Delta H{\text{ }}={H_{\left[ 6 \right]MCPP@gas~}} - \left( {{H_{MCPP}}+{H_{gas}}} \right)$$

7 $$\Delta S{\text{ }}={S_{\left[ 6 \right]MCPP@gas~}} - \left( {{S_{MCPP}}+{S_{gas}}} \right)$$

The electronic energy levels, namely the energy of the highest occupied molecular orbital (E_HOMO_) and the energy of the lowest unoccupied molecular orbital (E_LUMO_), were calculated to determine the energy gap (E_g_). Furthermore, density of states (DOS) analysis was performed using the GaussSum program^46^ to provide a detailed investigation of the electronic properties. Global reactivity descriptors, including chemical hardness (η), chemical potential (µ), electrophilicity index (ω), and the total amount of charge transfer (ΔN), were derived from E_HOMO_ and E_LUMO_ energies. These parameters were calculated using the following formulas^47^:

8 $$\eta =\frac{{{E_{LUMO}} - {E_{HOMO}}}}{2}$$

9 $$\mu =\frac{{{E_{LUMO}}+{E_{HOMO}}}}{2}$$

10 $$\omega ~=~\frac{{{\mu ^2}}}{{2\eta }}$$

11 $$\Delta N{\text{ }}=\frac{{\left( {{\mu _{\left[ 6 \right]MCPP~}} - ~{\mu _{gas}}} \right)~}}{{2\left( {{\eta _{\left[ 6 \right]MCPP~}}+~{\eta _{gas}}} \right)}}$$

The nature of the non-covalent interactions was characterized using the quantum theory of atoms in molecules (QTAIM), electron localization function (ELF), and non-covalent interaction (NCI) analyses, which were performed with the Multiwfn 3.8 software^48^. The corresponding three-dimensional NCI isosurfaces were visualized using the VMD program^49^. Furthermore, time-dependent density functional theory (TD-DFT) calculations at the same level of theory were conducted to simulate UV–vis spectra and analyze the excited-state properties.

## Results and discussion

### Stability of [6]MCPP nanobelt [6]MCPP@gas complexes

The structural stability of the free [6]MCPP nanobelt was evaluated by calculating its cohesive energy (E_coh_). Cohesive energy quantifies the strength of the bonding within a material, representing the energy required to dissociate it into its individual atoms. A more negative value signifies a more stable structure^50^. The calculated E_coh_ for the [6]MCPP nanobelt is -301.22 kcal/mol, confirming its high degree of structural stability. This significant negative cohesive energy indicates that the [6]MCPP nanobelt is energetically favorable and inherently resistant to structural decomposition. This robustness is a critical prerequisite for sensing materials, as it ensures the nanobelt can maintain its structural integrity during repeated adsorption and desorption cycles with target gas molecules.

**Fig. 2:**
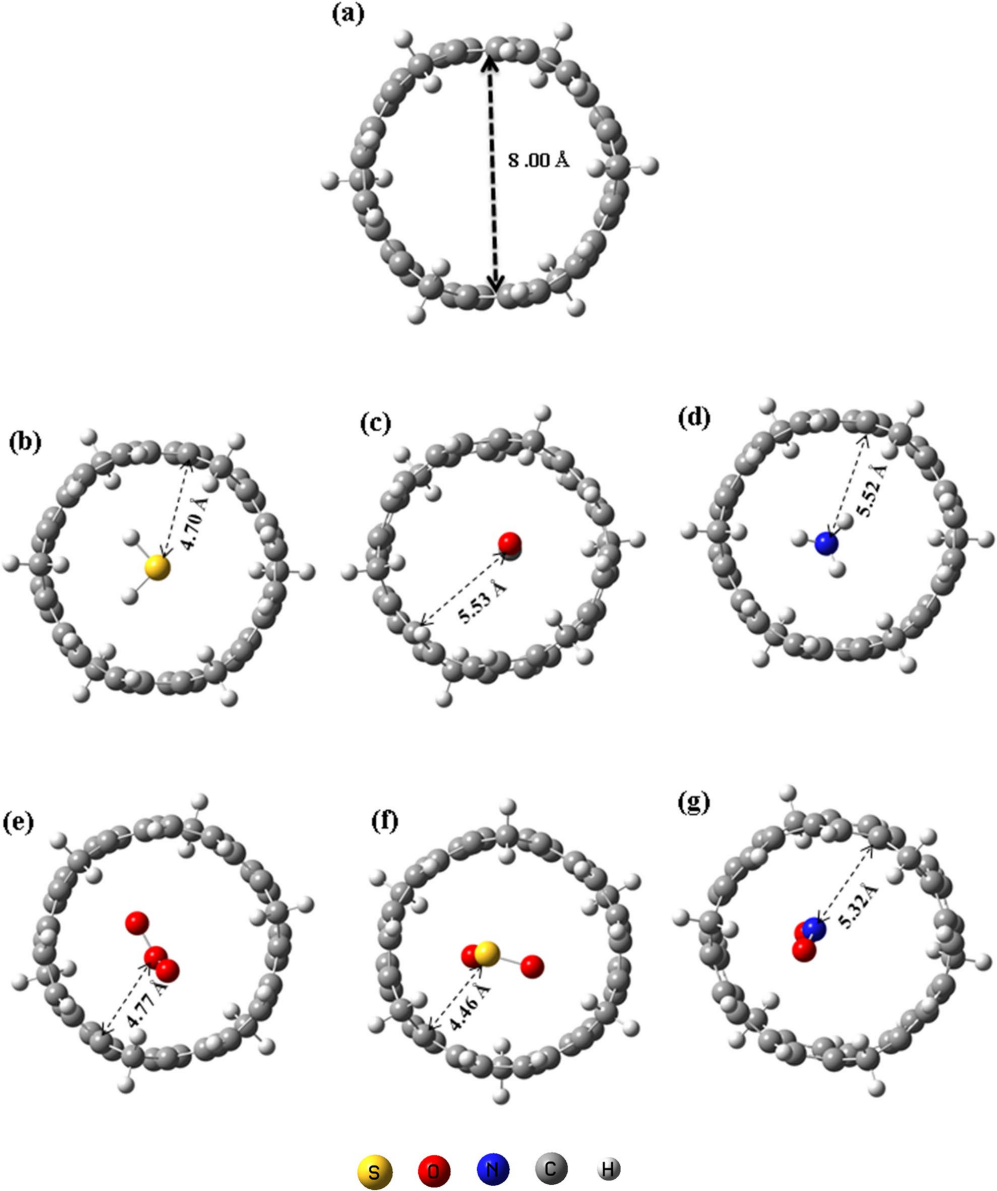
Optimized structures of (**a**) [6]MCPP, (**b**) [6]MCPP@H₂S, (**c**) [6]MCPP@CO, (**d**) [6]MCPP@NH₃, (**e**) [6]MCPP@O₃, (**f**) [6]MCPP@SO₂, and (**g**) [6]MCPP@NO₂.

The optimized geometries of the [6]MCPP@gas complexes are illustrated in Fig. 2. The porosity of the [6]MCPP nanobelt was estimated to be approximately 8 Å, providing suitable cavities for gas adsorption and diffusion. The interaction distances between the gas molecules and the nanobelt were found to be 4.70, 5.53, 5.52, 4.77, 4.46, and 5.32 Å for H_2_S, CO, NH_3_, O_3_, SO_2_, and NO_2_, respectively, depending on the type of adsorbed gas. Notably, the shortest interaction distance (4.46 Å) was observed for the [6]MCPP@SO₂ complex, indicating stronger electronic coupling and enhanced adsorption affinity toward SO_2_. Moreover, the analysis reveals no significant deformation in the [6]MCPP nanobelt structure upon interaction with any of the gas molecules, confirming its robust structural integrity and resilience. The interaction energies and key thermodynamic parameters for all complexes are summarized in Table 1. The negative interaction energies indicate that the adsorption process is energetically favorable for all the studied gas molecules. Among the examined systems, the [6]MCPP@SO₂ complex is the most stable, exhibiting the strongest interaction energy of − 20.68 kcal/mol, consistent with the shortest interaction distance. This suggests a high affinity for SO₂ and highlights the potential of [6]MCPP for selective sulfur dioxide detection. Besides, all [6]MCPP@gas complexes display small negative deformation energies (− 0.93 to − 1.02 kcal/mol), indicating that only minimal structural distortion occurs upon interaction and the nanobelt framework remains stable.

The thermodynamic analysis further reveals that the adsorption process is both spontaneous and exothermic for all complexes, as indicated by the negative values of the changes in Gibbs free energy (ΔG) and enthalpy (ΔH). The negative values of the entropy change (ΔS) indicate a decrease in disorder upon complex formation, which is expected when a gas molecule becomes constrained on the nanobelt surface. The fact that the reaction is driven by enthalpy (ΔH < 0) despite the unfavorable entropy loss (ΔS < 0) underscores the strength of the stabilizing interactions between [6]MCPP and the gas molecules.

**Table 1:** Interaction energy (E_int_), basis set superposition error (BSSE), counterpoise corrected interaction energy (E_,cp., int_), change in Gibbs free energy (ΔG), change in enthalpy (ΔH), change in entropy (ΔS), and deformation energy (E_def_) for [6]MCPP@gas complexes.

Complex	E_int_ (kcal/mol)	BSSE (kcal/mol)	E_int, cp._ (kcal/mol)	ΔH (kcal/mol)	ΔG (kcal/mol)	ΔS (kcal/mol K)	E_def_ (kcal/mol)
[6]MCPP@H₂S	− 15.75	1.91	− 13.84	− 15.81	− 7.65	− 0.027	− 0.95
[6]MCPP@CO	− 11.54	2.21	− 9.33	− 11.60	− 5.71	− 0.019	− 0.93
[6]MCPP@NH₃	− 12.80	2.03	− 10.77	− 12.86	− 8.84	− 0.013	− 0.99
[6]MCPP@O₃	− 20.58	3.36	− 17.22	− 20.51	− 11.23	− 0.064	− 1.02
[6]MCPP@SO₂	− 24.34	3.66	− 20.68	− 24.41	− 11.98	− 0.041	− 0.98
[6]MCPP@NO₂	− 14.49	2.26	− 12.23	− 14.49	− 7.09	− 0.024	− 0.98

### Electronic properties

Frontier molecular orbital (FMO) analysis was performed to evaluate the sensitivity, electronic structure, and reactivity of the [6]MCPP nanobelt upon interaction with gas molecules. A key parameter in this analysis is the energy gap (E_g_) between the highest occupied molecular orbital (HOMO) and the lowest unoccupied molecular orbital (LUMO). The electrical conductivity (σ) of a material is exponentially proportional to exp(− E_g_/2kT). Consequently, even a small reduction in E_g_ can lead to a significant enhancement in electrical conductivity. Therefore, a decreased HOMO–LUMO gap is directly associated with higher chemical reactivity, improved sensitivity, and superior charge transfer capabilities, which are essential for sensing applications. The percentage change in the energy gap (%ΔE_g_) due to gas adsorption was calculated using the following equation:

12 $$\% \Delta {E_g}=~\frac{{{E_g}\left( {complex} \right)~ - ~{E_g}\left( {\left[ 6 \right]MCPP} \right)}}{{{E_g}\left( {\left[ 6 \right]MCPP} \right)}}~ \times 100$$

Here, E_g_(complex) and E_g_([6]MCPP) refer to the energy gaps of the [6]MCPP@gas complexes and the free [6]MCPP nanobelt, respectively.

The calculated electronic properties, including E_HOMO_, E_LUMO_, E_g_, and %ΔE_g_, are summarized in Table 2. The E_g_ of the isolated [6]MCPP nanobelt is calculated to be 2.62 eV, which is in excellent agreement with the value of 2.66 eV reported by Kono et al.^24^, thereby validating our computational methodology. Upon interaction with gas molecules, the energy gap decreases for all [6]MCPP@gas complexes compared to the pristine nanobelt. This reduction in the HOMO–LUMO gap is directly linked to an exponential enhancement in electrical conductivity, which is a key factor for improved sensing sensitivity. Notably, the most significant reductions are observed for the [6]MCPP@O₃, [6]MCPP@SO₂, and [6]MCPP@NO₂ complexes (Table 2). These pronounced decreases suggest strong electronic coupling and charge transfer between the nanobelt and these specific gas molecules. Among all complexes, [6]MCPP@NO₂ exhibits the most dramatic change, with a remarkable %ΔE_g_ of − 67.17%. This substantial alteration underscores the superior electronic response of the nanobelt to NO₂. Such a significant band gap reduction not only greatly enhances the electrical conductivity but also reflects a strong affinity and efficient charge transfer mechanism between [6]MCPP and NO₂. These findings position [6]MCPP as a highly promising candidate for high-performance gas sensing, particularly for the detection of NO₂.

**Table 2:** Energy of HOMO (E_HOMO_), energy of LUMO (E_LUMO_), energy gap (E_g_), and percentage change in energy gap (%ΔE_g_) for [6]MCPP and [6] MCPP@gas complexes.

Compound	E_HOMO_ (eV)	E_LUMO_ (eV)	E_g_ (eV)	%ΔE_g_
[6]MCPP	− 4.52	− 1.90	2.62	**–**
[6]MCPP@H₂S	− 4.55	− 1.95	2.60	− 0.76
[6]MCPP@CO	− 4.50	− 1.89	2.61	− 0.38
[6]MCPP@NH₃	− 4.56	− 1.94	2.62	0.00
[6]MCPP@O₃	− 4.54	− 3.64	0.88	− 66.41
[6]MCPP@SO₂	− 4.52	− 3.43	1.09	− 58.39
[6]MCPP@NO₂	− 4.56	− 3.72	0.84	− 67.17

**Fig. 3:**
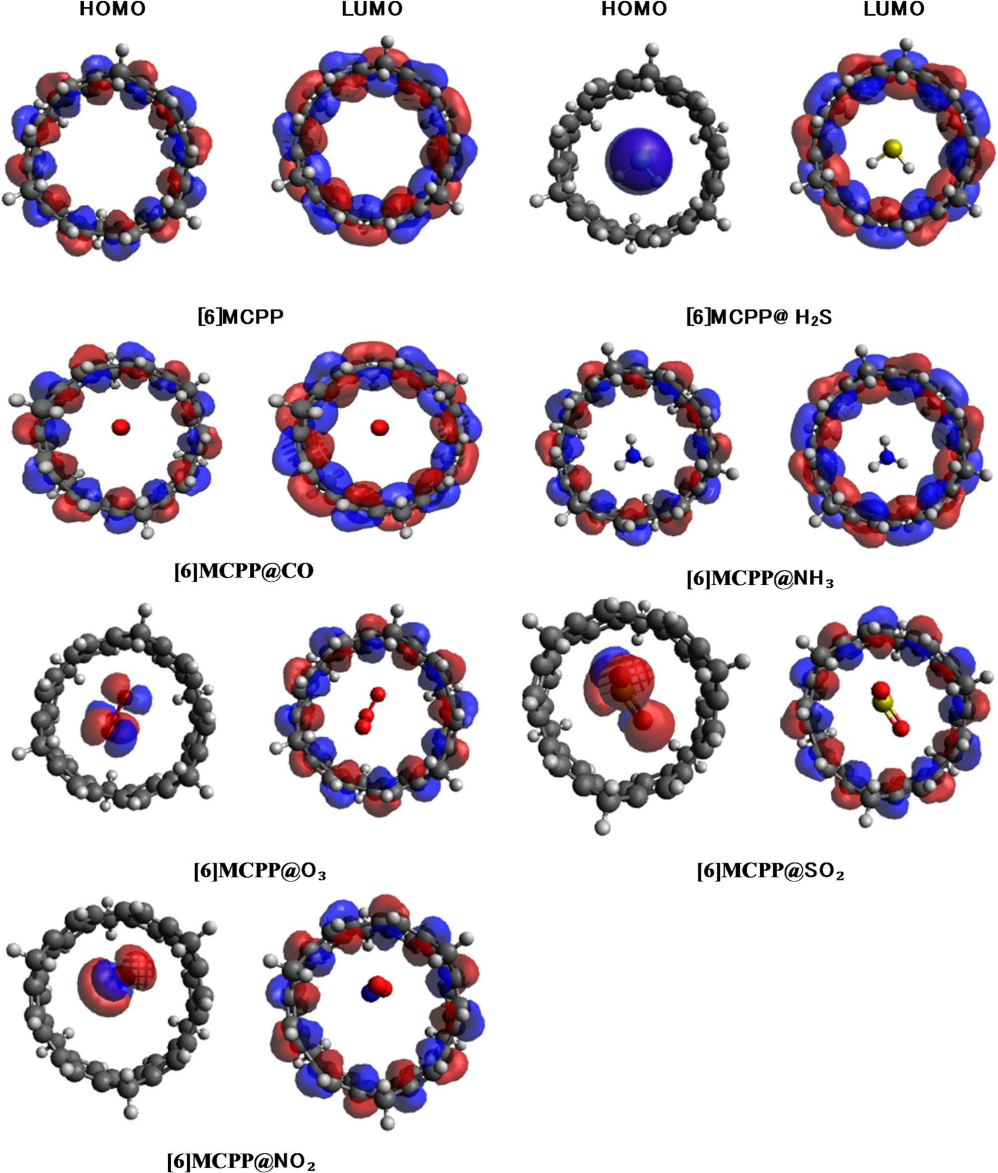
HOMO and LUMO diagrams for [6]MCPP and [6]MCPP@gas complexes.

The spatial distribution of the frontier molecular orbitals provides critical insight into the charge transfer behavior and electronic transitions within the [6]MCPP nanobelt upon gas adsorption, as depicted in Fig. 3. In the isolated [6]MCPP nanobelt, the HOMO and LUMO are uniformly delocalized across the entire π-conjugated framework. However, significant redistribution is observed upon interaction with gas molecules. A particularly notable pattern emerges for the [6]MCPP@O₃, [6]MCPP@SO₂, and [6]MCPP@NO₂ complexes, where a distinct spatial separation of orbitals occurs: the HOMO is primarily localized on the adsorbed gas molecule, while the LUMO remains concentrated on the [6]MCPP nanobelt. This specific orbital arrangement is a clear signature of efficient charge transfer (CT), indicating that electrons are readily excited from the HOMO (donor, gas molecule) to the LUMO (acceptor, nanobelt). This CT process facilitates strong electronic coupling and is a fundamental mechanism that enhances the electrical response and sensing capability of the material. The findings from these orbital diagrams are in excellent agreement with the DOS and energy gap analyses, demonstrating that the spatial separation of orbitals directly underpins the enhanced sensing performance. This consistent electronic behavior reinforces the potential of [6]MCPP as a highly responsive material for the selective detection of NO₂.

**Fig. 4:**
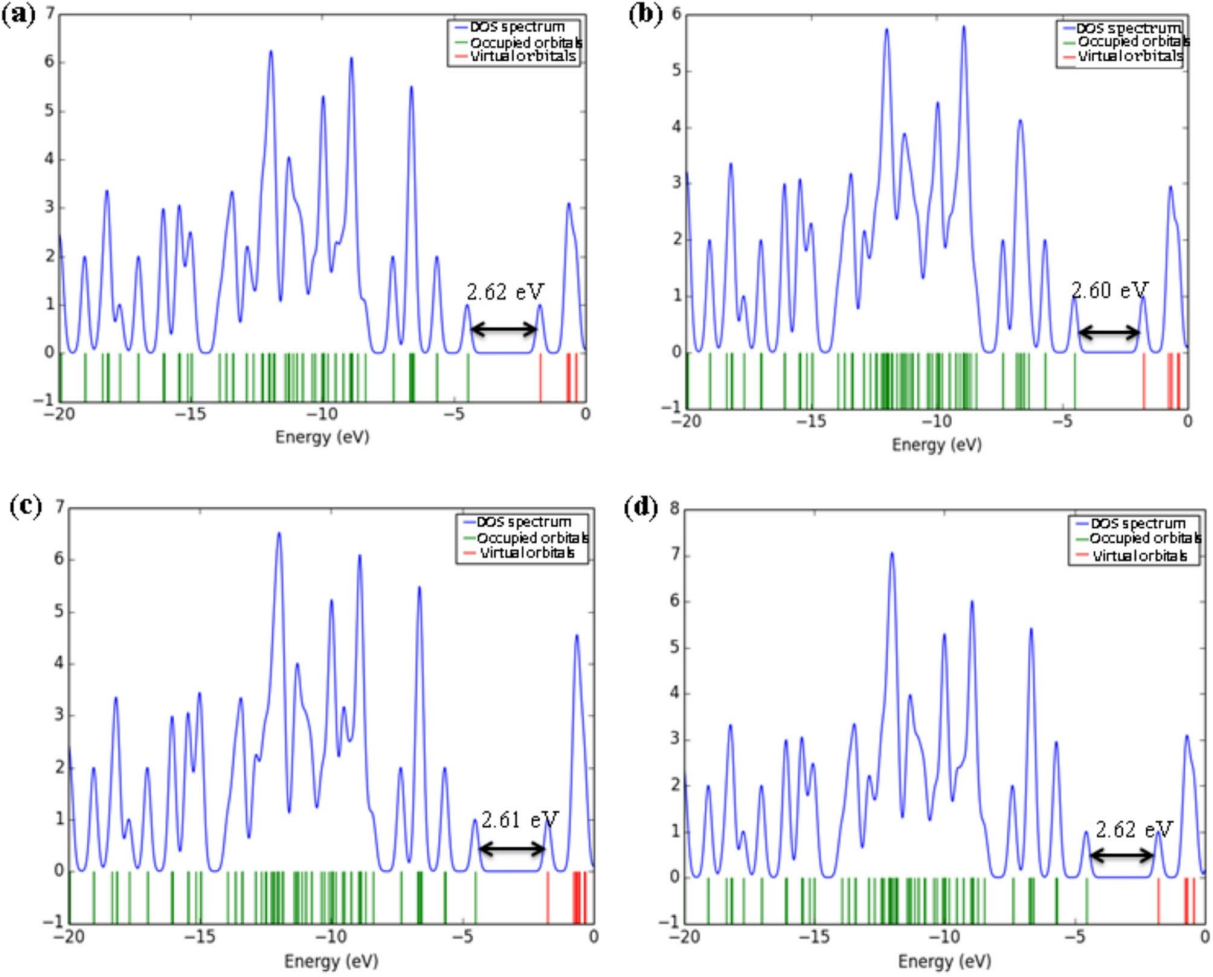

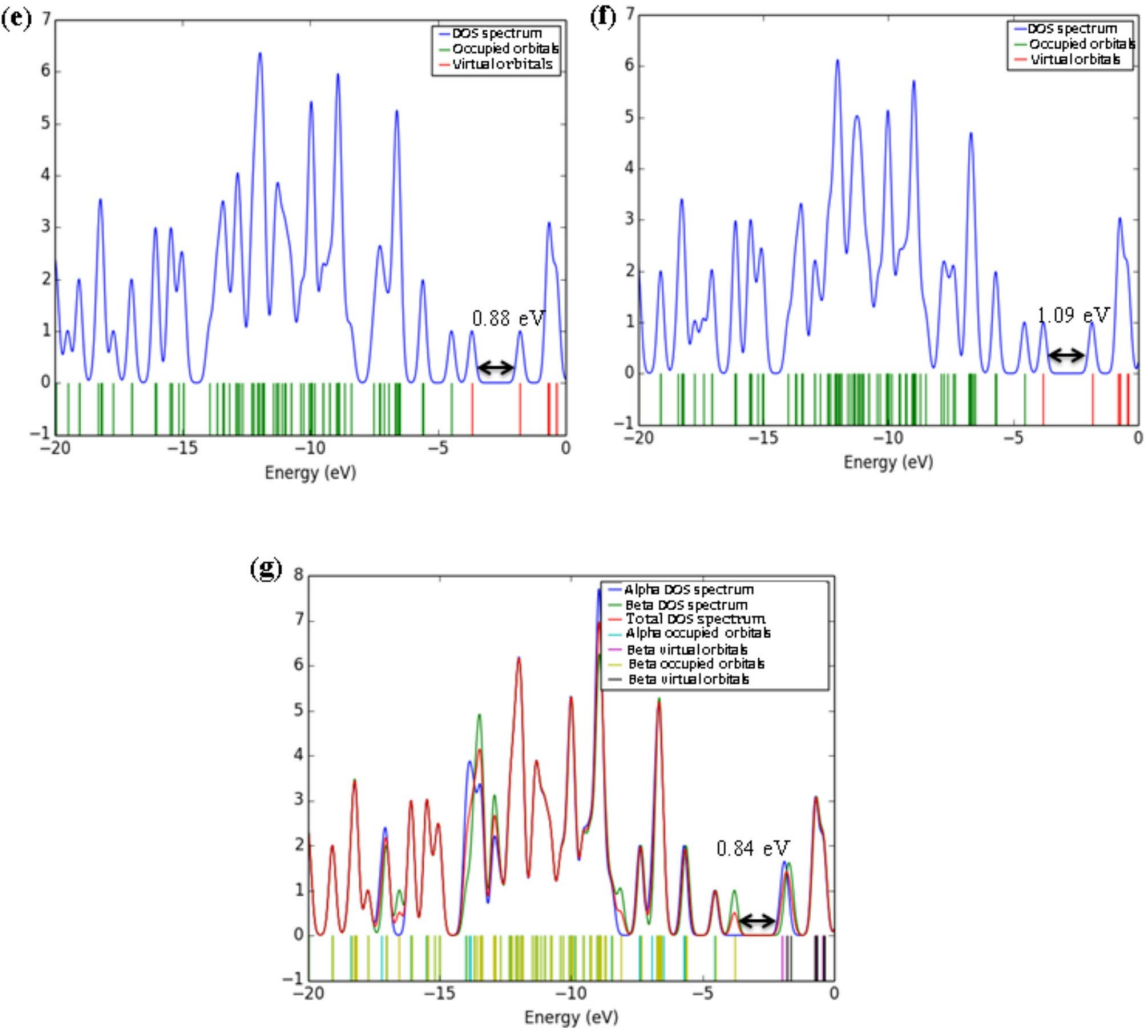
Graphical visualization of DOS spectra for (**a**) [6]MCPP, (**b**) [6]MCPP@H₂S, (**c**) [6]MCPP@CO, (**d**) [6]MCPP@NH₃, (**e**) [6]MCPP@O₃, (**f**) [6]MCPP@SO₂, and (**g**) [6]MCPP@NO₂.

Density of states (DOS) analysis provides detailed insight into the electronic structure of the [6]MCPP nanobelt and its modifications upon interaction with various gas molecules (Fig. 4). The DOS profile of the pristine [6]MCPP nanobelt shows a distinct energy gap of 2.62 eV, consistent with its moderate intrinsic conductivity and electronic stability. Significant alterations in the DOS profiles are observed upon formation of the gas complexes. The interactions with H₂S, CO, and NH₃ induce only minor perturbations near the Fermi level, suggesting weak physisorption and limited charge transfer. This finding aligns with their low sensitivity, as indicated by the interaction energy and %ΔE_g_ results.

In contrast, the complexes with O₃, SO₂, and NO₂ exhibit substantial modifications. These gases introduce new electronic states near the Fermi level, leading to a pronounced narrowing of the energy gap. The [6]MCPP@NO₂ complex demonstrates the most substantial effect, characterized by a dense population of states around the Fermi level. This indicates a dramatic reduction in the energy gap and a consequent marked increase in electrical conductivity, reflecting strong electronic coupling and efficient charge transfer between NO₂ and the nanobelt.

The observed changes in the electronic structure, particularly in the DOS spectra and frontier molecular orbitals upon gas adsorption, reveal the high sensitivity of the [6]MCPP nanobelt toward various gaseous species. These features suggest that [6]MCPP and its derivatives could serve as promising candidates for gas-sensing applications, especially for detecting toxic or environmentally hazardous gases such as NO₂, SO₂, and O₃. Moreover, the tunable electronic properties and structural stability of the [6]MCPP framework highlight its potential use in nanoelectronic and optoelectronic devices, as well as in molecular recognition and storage systems where selective adsorption and charge transfer play crucial roles.

The projected density of states (PDOS) analysis provides valuable insights into the electronic interactions between the [6]MCPP nanobelt and the adsorbed gas molecules. Among all the studied systems, the [6]MCPP@NO₂ complex exhibits the most significant orbital overlap between the molecular orbitals of the nanobelt and the NO₂ molecule near the Fermi level (see Figure S1). This pronounced overlap suggests a stronger electronic coupling and higher charge transfer efficiency compared to other gas complexes. The interaction leads to noticeable changes in the density of states around the HOMO–LUMO region, indicating enhanced hybridization between the π orbitals of [6]MCPP and the antibonding orbitals of NO₂. Such electronic modifications can improve the adsorption strength and sensitivity, making [6]MCPP particularly promising for NO₂ detection and sensing applications.

### Global reactivity indices

Global reactivity descriptors, including chemical potential (µ), chemical hardness (η), and electrophilicity index (ω), were employed to evaluate the influence of gas adsorption on the chemical reactivity and selectivity of the [6]MCPP nanobelt. The global indices for the isolated gas molecules are listed in Table S1, while those for the pristine [6]MCPP and the [6]MCPP@gas complexes are summarized in Table 3. As shown in Table 3, a significant decrease in chemical hardness (η) is observed for complexes with O₃, SO₂, and NO₂. According to Pearson’s hard-soft acid-base (HSAB) principle, this reduction indicates that these complexes are “softer” and thus more chemically reactive. A softer character enhances the polarizability of the system, making its electronic structure more adaptable and responsive to external stimuli, which is a crucial attribute for a sensing material. Furthermore, the consistently negative values of the chemical potential (µ) for all complexes confirm their enhanced thermodynamic stability after gas adsorption, underscoring their suitability for use in practical sensing environments. The analysis of these reactivity descriptors collectively suggests that the [6]MCPP nanobelt exhibits high sensitivity and strong reactivity, particularly toward O₃, SO₂, and NO₂. The charge transfer values (ΔN) provide further insight. The positive values of ΔN for all complexes signify that electron donation occurs from the nanobelt to the gas molecules. This confirms the role of [6]MCPP as an electron donor and the gas molecules (especially O₃, SO₂, and NO₂, which are strong electron acceptors) as electron acceptors in the interaction process. In conclusion, the observed charge transfer, combined with the enhanced reactivity, reduced chemical hardness, and maintained thermodynamic stability, demonstrates the significant potential of [6]MCPP for developing highly sensitive, selective, and reusable gas sensors capable of operating under realistic environmental conditions.

**Table 3:** Chemical hardness (η), chemical potential (µ), electrophilicity (ω), and total amount of charge transfer (ΔN) for [6]MCPP and [6]MCPP@gas complexes.

Compound	η (eV)	µ (eV)	ω(eV)	ΔN
[6]MCPP	1.31	− 3.21	3.93	–
[6]MCPP@H₂S	1.30	− 3.25	4.06	0.15
[6]MCPP@CO	1.30	− 3.19	3.91	0.18
[6]MCPP@NH₃	1.31	− 3.25	4.03	0.15
[6]MCPP@O₃	0.44	− 4.09	19.00	0.59
[6]MCPP@SO₂	0.54	− 3.97	14.59	0.47
[6]MCPP@NO₂	0.42	− 4.14	20.39	0.65

### UV–vis analysis

To gain deeper insight into the nature of the interactions, we conducted a detailed analysis of the UV-Vis absorption spectra for the pristine [6]MCPP nanobelt and its gas complexes (Figure S2). This analysis provides crucial information on the optical response and electronic transitions that occur upon gas adsorption. The key spectral parameters, maximum absorption wavelength (λ_max_), excitation energy (E_exc_), and oscillator strength (f), are summarized in Table 4. The pristine [6]MCPP nanobelt exhibits a strong absorption peak at 203.65 nm (E_exc_ = 6.08 eV) with an oscillator strength of 0.5, corresponding to its characteristic π→π* transitions. Adsorption of gas molecules induces noticeable changes in the spectra, most notably red-shifts in the absorption maxima. These red-shifts are consistent with a decrease in the HOMO-LUMO gap (as confirmed previously) and indicate a stabilization of the excited state due to enhanced charge-transfer interactions. The [6]MCPP@NO₂ complex demonstrates the most profound change, with a significant red-shift to λ_max_ = 333.45 nm (E_exc_ = 3.71 eV). This substantial decrease in excitation energy underscores a strong electronic coupling and substantial charge transfer between NO₂ and the nanobelt, corroborating the findings from the FMO and DOS analyses. These observations confirm that the optoelectronic properties of the nanobelt are highly sensitive to gas adsorption. The distinct and pronounced spectral shift exhibited by the [6]MCPP@NO₂ complex highlights the potential of [6]MCPP as a promising material for the selective and efficient photonic detection of NO₂ gas.

**Table 4:** Maximum absorption wavelength (λ_max_), excitation energy (E_exc_), Oscillation strength (f), and main transitions for [6]MCPP and [6]MCPP@gas complexes.

Complex	λ_max_ (nm)	E_exc_ (eV)	f	Main transitions
[6]MCPP	203.65	6.08	0.50	$\:\mathrm{H}-1\:\to\:\mathrm{L}+1\:\left(10\mathrm{\%}\right)\:\mathrm{H}\mathrm{O}\mathrm{M}\mathrm{O}\to\:\mathrm{L}+5\:\left(14\mathrm{\%}\right)$
[6]MCPP@H₂S	295.09	4.20	0.59	$\:\mathrm{H}-1\to\:\mathrm{L}\mathrm{U}\mathrm{M}\mathrm{O}\:\left(37\mathrm{\%}\right)\:\mathrm{H}\mathrm{O}\mathrm{M}\mathrm{O}\to\:\mathrm{L}+1\:\left(32\mathrm{\%}\right)$
[6]MCPP@CO	293.94	4.22	0.61	$\:\mathrm{H}-2\to\:\mathrm{L}\mathrm{U}\mathrm{M}\mathrm{O}\:\left(36\mathrm{\%}\right)\:\mathrm{H}\mathrm{O}\mathrm{M}\mathrm{O}\to\:\mathrm{L}+2\:\left(42\mathrm{\%}\right)$
[6]MCPP@NH₃	293.78	4.22	0.61	H-2$\:\to\:$LUMO (37%) HOMO$\:\to\:$L+2 (39%)
[6]MCPP@O₃	256.58	4.20	0.61	H-1$\:\to\:$LUMO (57%) HOMO$\:\to\:$L+1 (31%) HOMO$\:\to\:$L+2 (11%)
[6]MCPP@SO₂	297.63	4.16	0.58	H-2$\:\to\:$L+1 (16%) $\:\mathrm{H}-1\to\:\mathrm{L}+1\:\left(20\mathrm{\%}\right)\:\mathrm{H}\mathrm{O}\mathrm{M}\mathrm{O}\to\:\mathrm{L}+3\:\left(41\mathrm{\%}\right)$
[6]MCPP@NO₂	333.45	3.71	0.64	H-2$\:\to\:$L+1 (18%) H-1$\:\to\:$L+1 (20%) HOMO$\:\to\:$L+2 (40%)

### Electron density difference (EDD) analysis

**Fig. 5:**
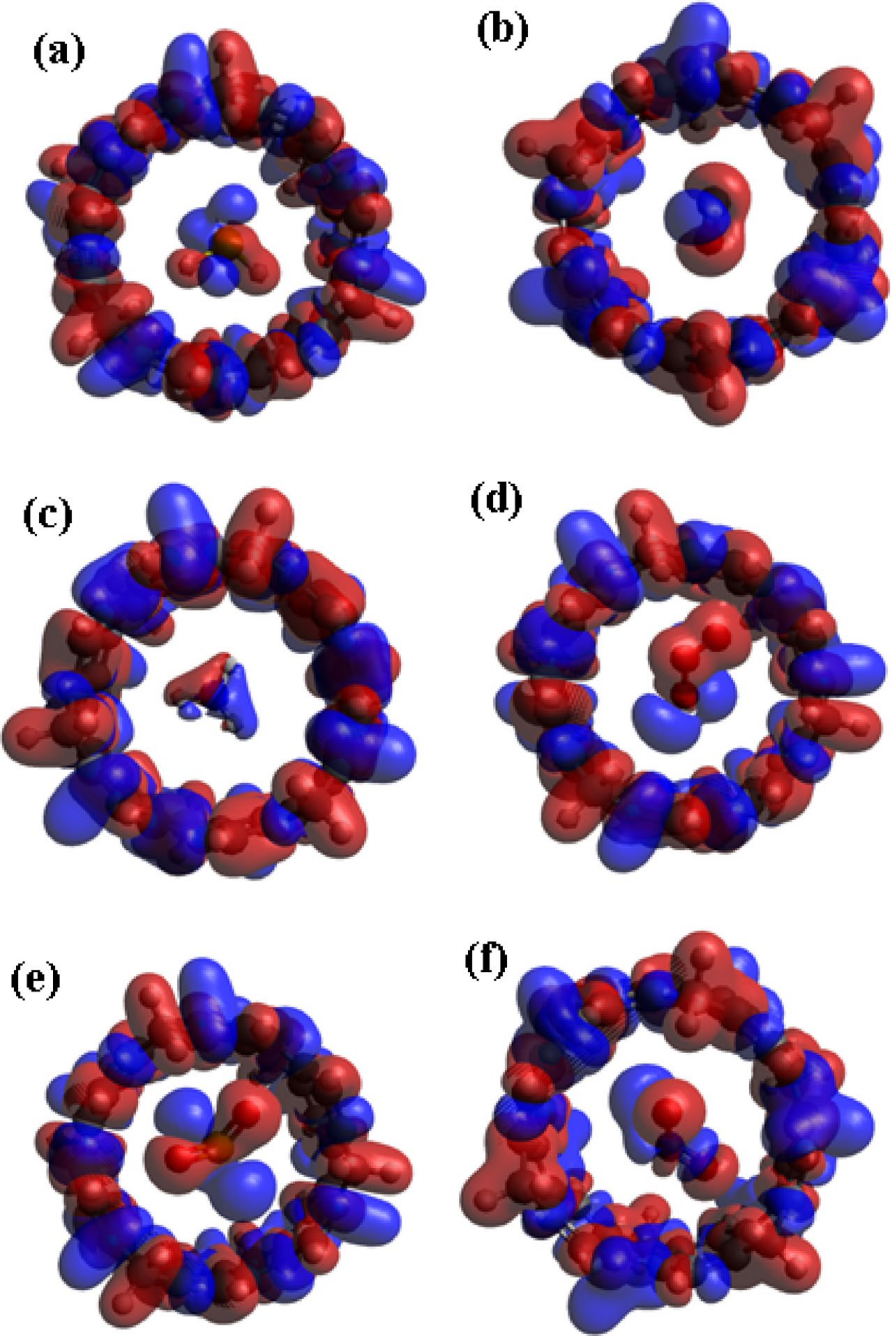
Representation of charge density difference (EDD) for (**a**) [6]MCPP@H₂S, (**b**) [6]MCPP@CO, (**c**) [6]MCPP@NH₃, (**d**) [6]MCPP@O₃, (**e**) [6]MCPP@SO₂, and (**f**) [6]MCPP@NO₂.

Electron density difference (EDD) analysis provides a detailed visual representation of charge redistribution between the [6]MCPP nanobelt and the adsorbed gas molecules upon complex formation. The EDD isosurfaces, generated by subtracting the sum of the electron densities of the isolated species from the density of the [6]MCPP@gas complex (Fig. 5), vividly illustrate regions of electron accumulation and depletion. In these maps, red isosurfaces indicate a gain in electron density, while blue isosurfaces represent a loss. The analysis reveals a consistent charge transfer mechanism for the most strongly interacting complexes ([6]MCPP@O₃, [6]MCPP@NO₂, and [6]MCPP@SO₂). The gas molecules are predominantly enveloped by red isosurfaces, indicating they are the primary sites of electron accumulation. Conversely, the adjacent regions of the nanobelt show blue isosurfaces, confirming it as the source of electron donation. This visual evidence of electron withdrawal by the gas molecules aligns perfectly with the positive charge transfer (ΔN) values calculated previously and provides a spatial context for the FMO results, which showed HOMO localization on the gases. The EDD analysis thus offers direct, visual confirmation of the charge transfer process, complementing the energetic data. Overall, the EDD analysis offers direct, visual confirmation of the charge transfer process, complementing the energetic and electronic data. These results collectively support the role of [6]MCPP as an effective electron donor for sensing strong electron-accepting gases and highlight its potential for high-performance gas-sensing applications.

### QTAIM analysis

**Fig. 6:**
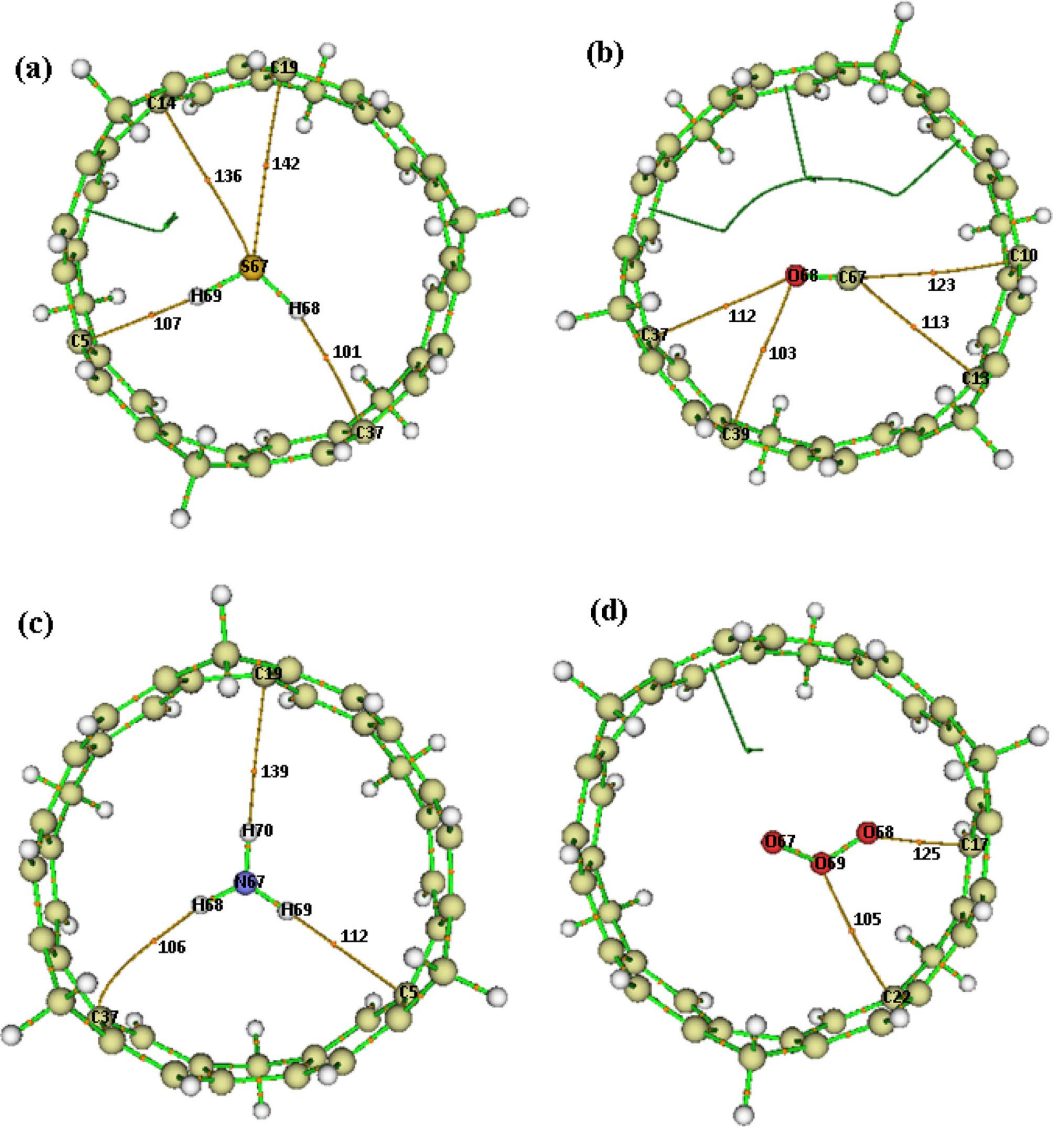

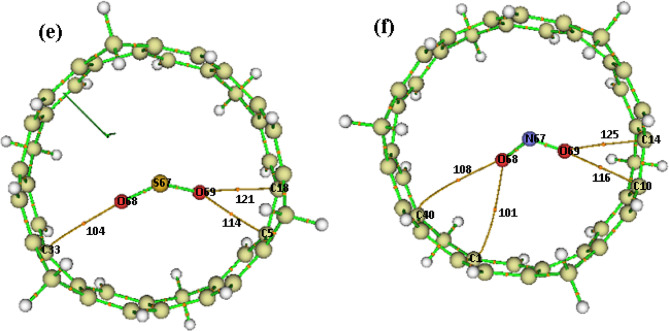
Quantum theory of atoms in molecule (QTAIM) molecular graphs for [6]MCPP@gas complexes. The BCPs are shown with orange points (**a**) [6]MCPP@H2S, (**b**) [6]MCPP@CO, (**c**) [6]MCPP@NH3, and (**d**) [6]MCPP@O3, (**e**) [6]MCPP@SO2, and (**f**) [6]MCPP@NO2.

In the QTAIM analysis, several key parameters were evaluated at the bond critical points (BCPs) to characterize the intermolecular interactions, including the electron density (ρ(r)), the Laplacian of electron density (∇²ρ(r)), kinetic energy density (G(r)), potential energy density (V(r)), and total energy density (H(r)). The identified BCPs are illustrated in the topological graphs in Fig. 6, and the corresponding parameters are summarized in Table 5. The strength of the interactions can be inferred from the ρ(r) values. Values greater than 0.1 a.u. typically indicate strong covalent bonding, whereas values below 0.1 a.u. suggest weaker, non-covalent interactions^51^. As shown in Table 5, the ρ(r) values for all complexes fall within the range of 0.02 to 0.08 a.u., confirming the predominance of weak non-covalent interactions.

All complexes exhibit positive values for both ∇²ρ(r) and H(r), which is a hallmark of closed-shell (non-covalent) interactions. The ratio of − G(r)/V(r) provides further insight into the nature of these interactions. According to the criteria established by Espinosa et al.^52^, a ratio greater than 1 is characteristic of pure non-covalent interactions, values between 0.5 and 1 imply electrostatic interactions with partial covalent character, and values below 0.5 indicate covalent bonding. For all studied complexes, the − G(r)/V(r) ratios were consistently greater than 1, unequivocally confirming the purely non-covalent nature of the adsorption. Overall, the QTAIM findings corroborate the earlier electronic and energetic analyses, demonstrating that gas adsorption on the [6]MCPP nanobelt is governed predominantly by weak, non-covalent interactions. This mechanism is highly desirable as it reinforces the material’s potential for fully reversible gas sensing applications, enabling sensor regeneration.

**Table 5:** Topological parameters (in a.u.) at BCP for intermolecular interactions in [6]MCPP@gas complexes.

Complex	BCP	Bond	ρ(*r*)	$\:{\nabla\:}^{2}{\uprho\:}$(*r*)	H(*r*)	G(*r*)	V(*r*)	− G(*r*)/V(*r*)
[6]MCPP@H₂S	101 107 136 142	H68$\:-$C37 H69$\:-$C5 S67$\:-$C14 S67$\:-$C19	0.05 0.07 0.02 0.02	0.19 0.30 0.08 0.07	0.01 0.01 − 0.005 − 0.004	0.04 0.06 0.015 0.014	− 0.03 − 0.05 − 0.011 − 0.01	1.33 1.20 1.36 1.40
[6]MCPP@CO	103 112 113 123	O68$\:-$C39 O68$\:-$C37 O67$\:-$C10 H67$\:-$C13	0.02 0.03 0.04 0.03	0.12 0.15 0.15 0.13	0.005 0.007 0.008 0.007	0.024 0.031 0.03 0.026	− 0.019 − 0.024 − 0.022 − 0.019	1.26 1.29 1.36 1.37
[6]MCPP@NH₃	106 112 139	H68$\:-$C37 H69$\:-$C5 H70$\:-$C19	0.03 0.04 0.02	0.12 0.15 0.09	0.008 0.009 0.003	0.024 0.029 0.013	− 0.016 − 0.02 − 0.01	1.50 1.45 1.30
[6]MCPP@O₃	105 125	O69$\:-$C22 O68$\:-$C17	0.05 0.03	0.21 0.16	0.007 0.003	0.047 0.038	− 0.04 − 0.035	1.17 1.05
[6]MCPP@SO₂	104 114 121	O68$\:-$C33 O69$\:-$C5 O69$\:-$C18	0.08 0.02 0.02	0.40 0.10 0.10	0.014 0.003 0.003	0.086 0.022 0.023	− 0.072 − 0.019 − 0.020	1.19 1.15 1.15
[6]MCPP@NO₂	101 108 116 125	O68$\:-$C1 O68$\:-$C40 O69$\:-$C10 O69$\:-$C14	0.04 0.04 0.08 0.08	0.14 0.15 0.45 0.48	0.006 0.006 0.004 0.003	0.03 0.033 0.018 0.019	− 0.024 − 0.027 − 0.014 − 0.016	1.25 1.22 1.28 1.18

### Non-covalent interaction (NCI) analysis

Non-covalent interaction (NCI) analysis was employed to visualize and characterize the closed-shell interactions between the gas molecules and the [6]MCPP nanobelt using both 2D reduced density gradient (RDG) scatter plots and their corresponding 3D isosurfaces, as illustrated in Figure S3. In this analysis, the type of interaction is denoted by color on a sign(λ₂)ρ map: blue regions (sign(λ₂)ρ < 0) indicate strong attractive interactions such as hydrogen bonding or electrostatic attraction, green regions (sign(λ₂)ρ ≈ 0) represent weak van der Waals forces, and red regions (sign(λ₂)ρ >0) signify strong repulsive or steric effects^53^. For the studied complexes, green isosurfaces predominantly appear between the gas molecules and the nanobelt, confirming that the interactions are primarily mediated by van der Waals forces. Notably, the [6]MCPP@O₃, [6]MCPP@SO₂, and [6]MCPP@NO₂ complexes also exhibit distinct blue isosurfaces, indicating the presence of stronger attractive interactions alongside the van der Waals forces. This is further supported by the green and blue spikes observed in the 2D RDG plots, which confirm the coexistence of both interaction types. Additionally, red spikes in the RDG plots within the sign(λ₂)ρ range of 0.01 to 0.05 a.u. correspond to red patches on the 3D isosurfaces located within the aromatic rings of the nanobelt, indicating steric hindrance within the conjugated π-system. These NCI findings provide crucial visual evidence that the adsorption is primarily physisorptive, a desirable feature for sensor reversibility, while the stronger attractive components in O₃, SO₂, and NO₂ complexes explain their enhanced sensitivity and selectivity.

### Electron localization function (ELF) analysis

To further elucidate the nature of the interactions between gas molecules and the [6]MCPP nanobelt, the electron localization function (ELF) was computed, and the results are presented in Figure S4. The ELF describes the probability of finding an electron in the neighborhood of a reference electron with the same spin, ranging from 0 to 1, where ELF ≈ 0 (blue regions) indicates fully delocalized electrons (typical of non-bonding or weak van der Waals interactions), and ELF ≈ 1 (red regions) represents strong electron localization associated with covalent bonding. Intermediate green–yellow regions correspond to moderately localized electrons, as seen in ionic or polar interactions. As illustrated in Figure S4, the ELF maps reveal that the regions between the gas molecules and the [6]MCPP surface are predominantly blue, confirming low electron localization and the absence of significant charge accumulation between interacting atoms. This behavior indicates that no covalent bonds are formed between the adsorbed gases and the nanobelt. Instead, the interaction is governed mainly by weak non-covalent forces, such as van der Waals and electrostatic interactions. Among all the systems studied, the [6]MCPP@NO₂ complex exhibits slightly enhanced electron localization at the adsorption interface, consistent with its higher adsorption energy and stronger charge transfer. However, the ELF still remains below 0.5, suggesting that even in this case, the interaction is physisorptive rather than chemisorptive.

### Sensing performance

#### Electrical conductivity

The electrical conductivity (σ) of a material is fundamentally governed by the ease with which electrons can transition from the valence band to the conduction band. This electron promotion is quantitatively related to the energy gap (E_g_) between these states. As expressed in Eq. (13), electrical conductivity exhibits an inverse exponential relationship with the energy gap^54^. Consequently, even minor modifications to the electronic structure of the [6]MCPP nanobelt, such as those induced by gas adsorption, can profoundly alter its electrical resistivity and conductivity. The observed significant reduction in E_g_ for certain complexes, therefore, directly predicts a substantial enhancement in their electrical conductivity, which is the fundamental mechanism underlying the sensing response.

13 $$\sigma \,=\,{\mathrm{A}}{{\mathrm{T}}^{{\mathrm{3}}/{\mathrm{2}}}}{\mathrm{exp}}\left( {\frac{{ - {{\mathrm{E}}_{\mathrm{g}}}}}{{2{{\mathrm{k}}_{\mathrm{B}}}{\mathrm{T}}}}} \right)$$

In this equation, A is the Richardson constant (6 × 10⁵ A m^−2^), T is the temperature (298 K), and K_B_ is the Boltzmann constant (1.38 × 10^–23^ J/K). The inverse exponential relationship dictates that the lower energy gaps observed in the [6]MCPP@gas complexes result in a significant enhancement of their electrical conductivity, which forms the fundamental mechanism underlying the sensing response. The calculated conductivity values for all complexes are summarized in Table 6. Among them, the [6]MCPP@NO₂ complex exhibits the highest electrical conductivity of 239 S/m, corresponding to its exceptionally low energy gap of 0.84 eV. This pronounced increase in conductivity provides quantitative evidence that the [6]MCPP nanobelt is a highly promising candidate for high-performance gas sensing, with exceptional sensitivity and efficiency toward NO₂ detection.

#### Sensing response

The sensing response (s) plays a crucial role in determining the sensitivity and detection range of gas molecules in the investigated complexes, which is evaluated based on the following Eq. 5^2^:

14 $${\text{S }}=\frac{{{{{{\upsigma}}}_{{\mathrm{Complex}}}}}}{{{{{{\upsigma}}}_{\left[ 6 \right]{\mathrm{MCPP}}}}}} - 1$$

**Fig. 7:**
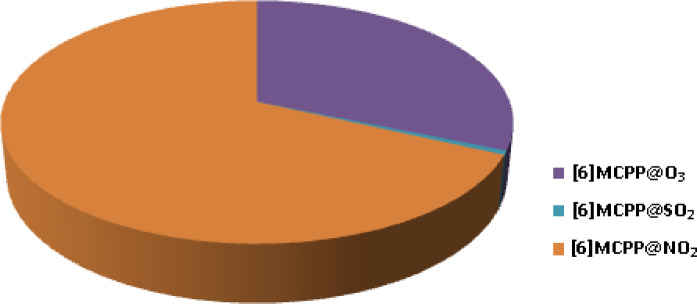
Pie chart showing the considerable sensitivity distribution of [6]MCPP@gas complexes.

Here, σ_complex,_ and σ_[6]MCPP_ represent the electrical conductivities of the [6]MCPP@gas complexes and the pristine [6]MCPP nanobelt, respectively. Electrical conductivity is a key factor influencing the sensitivity of the sensing system, with higher conductivity typically indicating greater sensitivity. As shown in Table 6, the sensitivity values reveal that among all the studied complexes, [6]MCPP@O₃, [6]MCPP@SO₂, and [6]MCPP@NO₂ exhibit notable sensing responses of 3.36 × 10^14^, 5.596 × 10^12^, and 7.30 × 10^14^, respectively. These results confirm that O₃, SO₂, and NO₂ gas molecules are effectively detected by the [6]MCPP nanobelt. In addition, Fig. 7 presents a pie chart illustrating the percentage of sensing responses of the complexes. This chart provides a clear visual comparison of the relative sensing performance of each complex, with values expressed as percentages of the total response. A higher value corresponds to a stronger sensing capability. Among the systems investigated, the [6]MCPP@NO₂ complex demonstrates the highest sensitivity, accounting for 68% of the total response. This significant variation in sensing performance highlights the exceptional potential of the [6]MCPP nanobelt as a selective, sensitive, and versatile material for gas sensing applications.

#### Work function

The work function (φ) represents the minimum energy required to remove an electron from the Fermi level of a material and transfer it to the vacuum. As a surface-sensitive property, the work function can significantly influence the material’s electrical conductivity. It is related to the Fermi energy (E_F_) through the following relationship^55^:

15 $$\varphi {\text{ }}=\left| {{V_\infty } - {E_F}} \right|$$

Here, V_∞_ ​ represents the vacuum potential. In practical calculations, this potential is often set as the reference point and taken to be zero. The calculated work functions (see Table 6) indicate that [6]MCPP@NO₂ exhibits the highest value of 4.14 eV, reflecting the strongest electronic perturbation among the studied complexes and suggesting its superior sensing capability.

#### 1.2.1. Recovery time

The recovery time (τ) denotes the period required for the analyte to desorb from the substrate, playing a key role in evaluating the sensitivity and reusability of sensing materials. A strong chemical interaction between the analyte and substrate typically leads to an extended recovery time. Based on transition state theory^56^, the recovery time can be calculated using the following equation:

16 $$\tau \,=\,\upsilon _{0}^{{ - 1}}exp\left( {\frac{{ - {E_{int}}}}{{{K_B}T}}} \right)$$

In this equation, υ₀ represents the attempt frequency, typically assigned a value of 10¹² s^− 1^. The recovery time exhibits an exponential dependence on the interaction energy; as the interaction energy becomes more negative (stronger binding), the recovery time increases exponentially. Consequently, a lower magnitude of interaction energy (less negative value) indicates a more reversible adsorption process and is highly desirable for sensor regeneration. The calculated recovery times for all complexes at 298 K are presented in Table 6. The notably short recovery times observed for the [6]MCPP@CO, [6]MCPP@NH₃, and [6]MCPP@NO₂ complexes suggest that these gas molecules can desorb readily from the nanobelt surface. This highlights the material’s potential for fast sensor response and excellent reusability.

Although the [6]MCPP@CO complex exhibits the shortest recovery time (7.18 × 10^− 6^ s), NO₂ emerges as the most promising target analyte. Despite its strong interaction and high sensitivity, evidenced by a significant charge transfer and band gap reduction, NO₂ maintains a relatively short recovery time of 9.78 × 10^− 4^ s, which is much lower than previous reports^57–59^ This optimal balance between strong adsorption (high sensitivity) and facile desorption (fast recovery) underscores the exceptional sensing performance of the [6]MCPP nanobelt for NO₂ detection, positioning it as a highly efficient and selective material for practical, reusable gas sensing applications.

**Table 6:** Electrical conductivity (σ), sensing response, work function (φ), and recovery time (τ) for [6]MCPP and [6]MCPP@gas complexes.

Compound	σ (S/m)	S	φ (eV)	τ (s)
[6]MCPP	3.27 $\:\times\:$ 10^–13^	**–**	3.21	**–**
[6]MCPP@H₂S	3.88 $\:\times\:\:$10^–13^	0.18	3.25	1.45 × 10^− 2^
[6]MCPP@CO	3.55$\:\:\times\:$ 10^–13^	0.08	3.19	7.18 × 10^− 6^
[6]MCPP@NH₃	3.27 $\:\times\:$ 10^–13^	0	3.25	1.10 × 10^− 4^
[6]MCPP@O₃	110.00	3.36 × 10^14^	4.09	5.19
[6]MCPP@SO₂	1.83	5.59 × 10^12^	3.97	1.56 × 10^3^
[6]MCPP@NO₂	239.00	7.30 × 10^14^	4.14	9.78 × 10^− 4^

## Conclusion

In this study, the gas-sensing performance of the methylene-bridged [6]cycloparaphenylene ([6]MCPP) nanobelt was systematically investigated for toxic gases (H₂S, CO, NH₃, O₃, SO₂, and NO₂) using density functional theory (DFT) calculations. The nanobelt exhibited remarkable structural stability, as evidenced by its high cohesive energy of -301.22 kcal/mol, confirming its suitability for durable and reusable sensing applications. Among the investigated gases, NO₂ displayed the most promising interaction with [6]MCPP, characterized by a substantial reduction in the HOMO–LUMO energy gap (67.17%), indicating high sensitivity, a moderate interaction energy (− 12.33 kcal/mol), reflecting an optimal balance between adsorption strength and desorption feasibility, and a short recovery time (9.78 × 10⁻⁴ s), enabling rapid sensor regeneration. In contrast, SO₂ exhibited the strongest interaction energy (-20.68 kcal/mol), which, although indicative of high affinity, may hinder desorption and compromise reusability. Analyses based on QTAIM, ELF, and NCI consistently demonstrated that adsorption is predominantly governed by non-covalent interactions, particularly van der Waals forces, ensuring reversibility, while the stronger attractive components in the [6]MCPP@O₃, [6]MCPP@SO₂, and [6]MCPP@NO₂ complexes account for their enhanced sensitivity and selectivity. Overall, the [6]MCPP nanobelt emerges as a highly promising candidate for next-generation gas sensors, achieving an ideal combination of high sensitivity, excellent selectivity (especially toward NO₂), robust structural integrity, and rapid recovery, positioning it as an efficient material for practical environmental monitoring and toxic gas detection applications.

**Funding Declaration**.

No funding was required for this study.

## Supplementary Information

Below is the link to the electronic supplementary material.

Supplementary Material 1

## Data Availability

Data sets generated during the current study are available from the corresponding author (Rima Heider Al Omari) upon reasonable request.
