# Supplementary material for: Unveiling the gas-sensing potential of methylene-bridged [6]cycloparaphenylene nanobelt: a DFT perspective
Source: Sci Rep. 2025 Nov 23;15:44614. doi: 10.1038/s41598-025-28684-1 (PMC12738587; doi:10.1038/s41598-025-28684-1)
Supplement: Supplementary file 1 — Supplementary Material 1 [file 41598_2025_28684_MOESM1_ESM.docx]

**Supporting Information**

**Unveiling the Gas-Sensing Potential of Methylene-Bridged [6]Cycloparaphenylene Nanobelt: A DFT Perspective**

Rima Heider Al Omariᵃ^*^, Shelesh Krishna Saraswatᵇ, Munthar Kadhim Abosaodaᶜ,ᵈ, Rekha M. M.ᵉ, Subhashree Ray^f^, Kattela Chennakesavulu^g^, Renu Sharma^h^, Aditya Kashyap^k^

^a^ Department of Medical Laboratory Sciences, Faculty of Allied Medical Sciences, Hourani Center for Applied Scientific Research, Al-Ahliyya Amman University, Amman, Jordan
ᵇ Department of Electronics and Communication Engineering, GLA University, Mathura-281406, India
ᶜ College of Pharmacy, The Islamic University, Najaf, Iraq
ᵈ College of Pharmacy, The Islamic University of Al Diwaniyah, Al Diwaniyah, Iraq
ᵉ Department of Chemistry and Biochemistry, School of Sciences, JAIN (Deemed-to-be University), Bangalore, Karnataka, India

^f^ Department of Biochemistry, IMS and SUM Hospital, Siksha 'O' Anusandhan (Deemed to be University), Bhubaneswar, Odisha-751003, India,
^g^ Department of Chemistry, Sathyabama Institute of Science and Technology, Chennai, Tamil Nadu, India
^h^ Department of Chemistry, University Institute of Sciences, Chandigarh University, Mohali, Punjab, India
^k^ Centre for Research Impact & Outcome, Chitkara University Institute of Engineering and Technology, Chitkara University, Rajpura-140401, Punjab, India

^*^[Rima.H.AA@proton.me](mailto:Rima.H.AA@proton.me)

| **Table S1.**  Energy of HOMO (E_HOMO_), energy of LUMO (E_LUMO_), energy gap (E_g_), Chemical hardness (η), and chemical potential (μ) of gas molecules. | | | | | |
| --- | --- | --- | --- | --- | --- |
| **Compound** | **E_HOMO_ (eV)** | **E_LUMO_ (eV)** | **E_g_ (eV)** | **η (eV)** | **μ (eV)** |
| H_2_S | -7.20 | -0.91 | 6.29 | 3.14 | -4.05 |
| CO | -10.03 | -0.69 | 9.34 | 4.67 | -5.36 |
| NH_3_ | -5.83 | -2.45 | 3.38 | 1.69 | -4.14 |
| O_3_ | -9.03 | -5.14 | 3.89 | 1.94 | -7.08 |
| SO_2_ | -8.88 | -4.31 | 4.57 | 2.28 | -6.59 |
| NO_2_ | -9.04 | -5.50 | 3.54 | 1.77 | -7.27 |


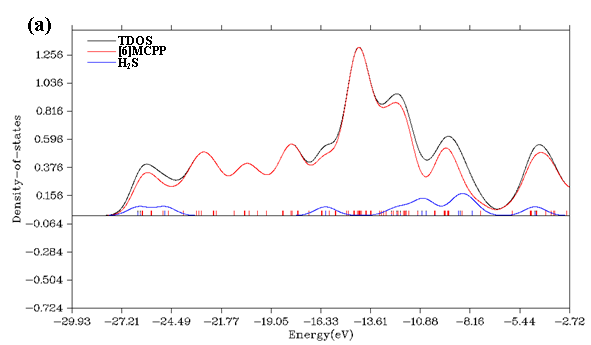


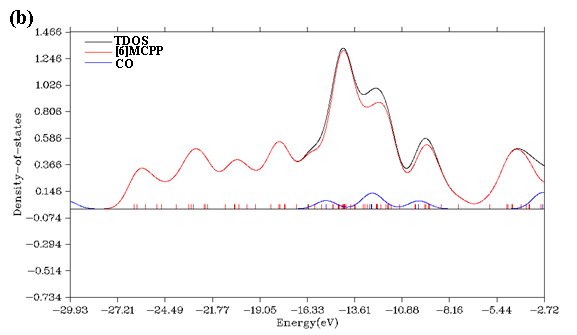


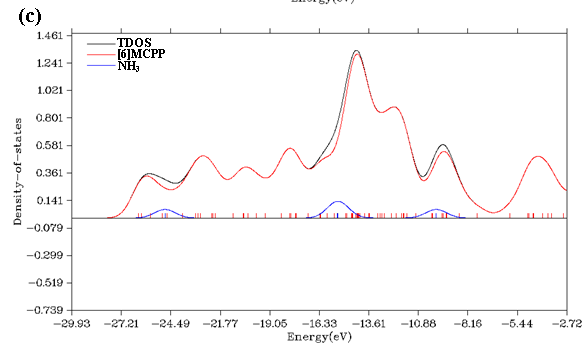


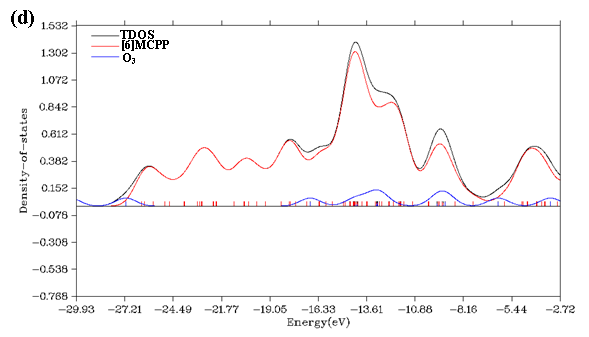


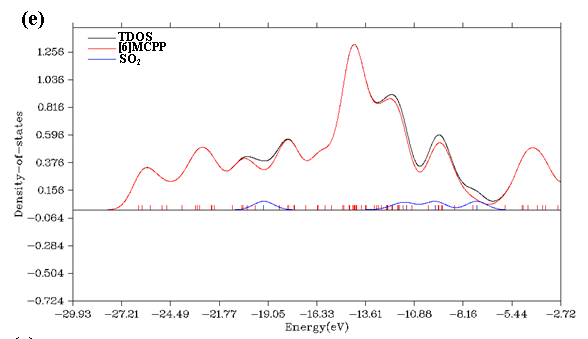


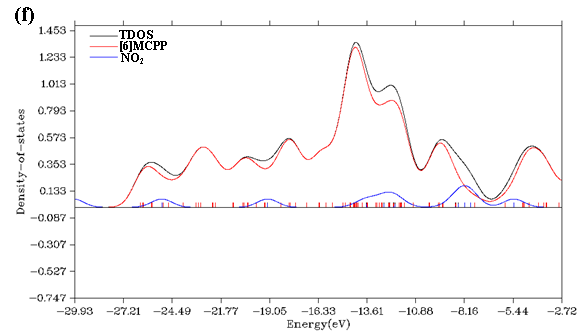


**Figure S1.** Graphical visualization of PDOS spectra for (a) [6]MCPP@H₂S, (b) [6]MCPP@CO, (c) [6]MCPP@NH₃, (d) [6]MCPP@O₃, (e) [6]MCPP@SO₂, and (f) [6]MCPP@NO₂.


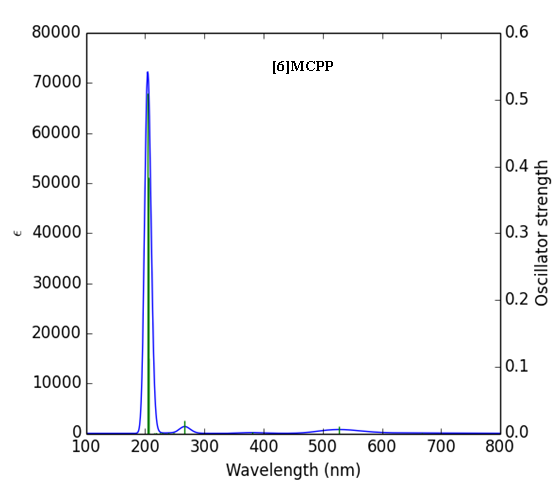


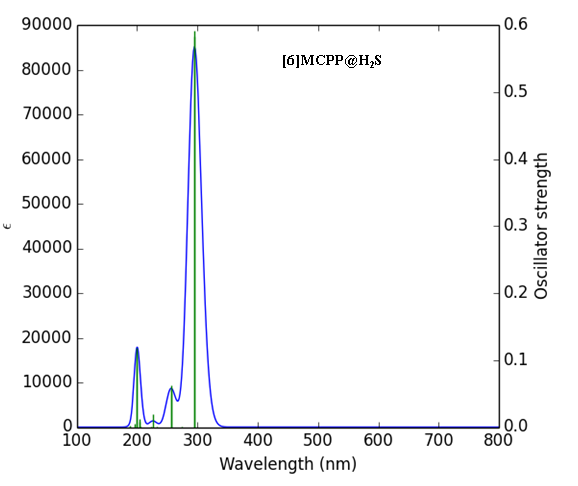


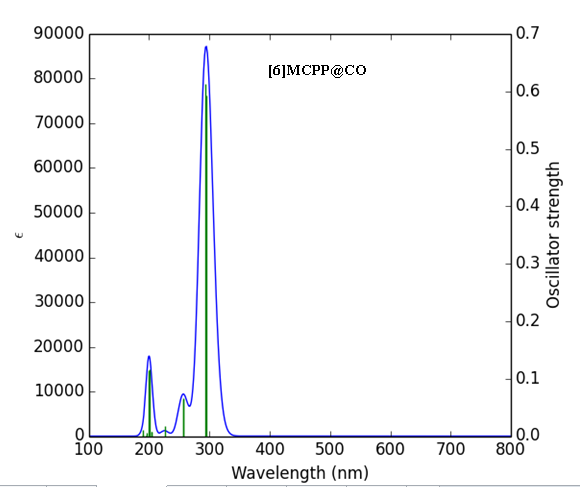


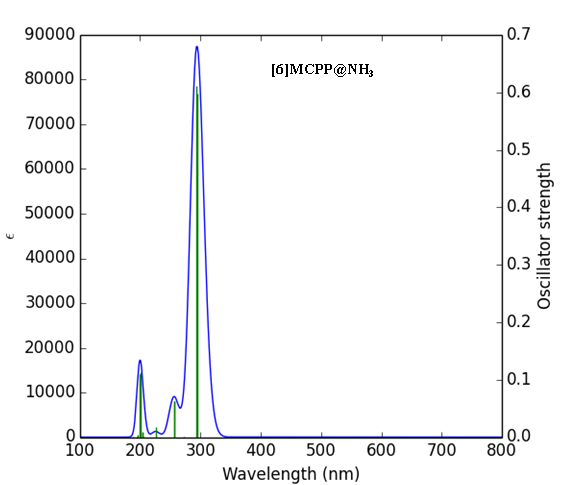


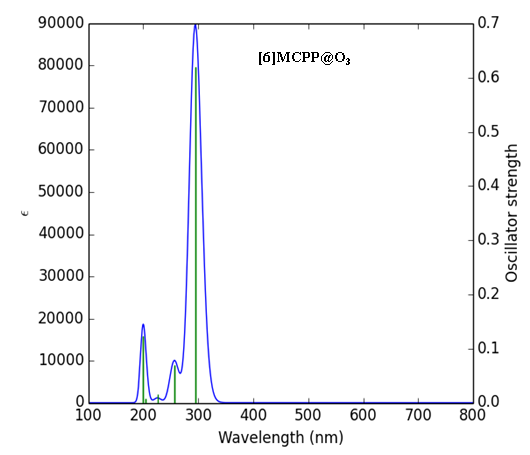


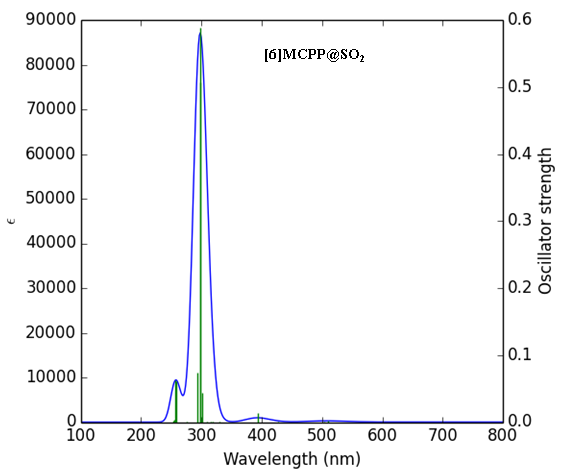


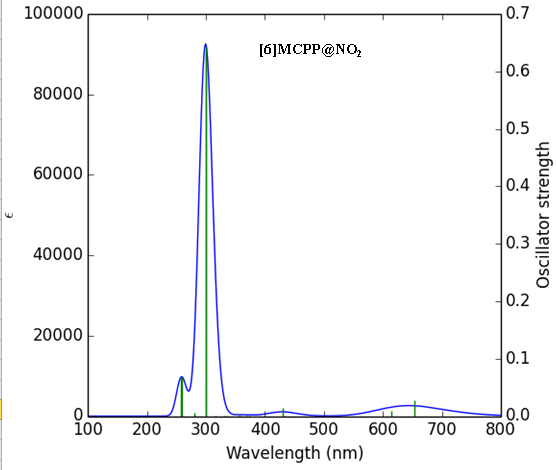


**Figure S2.** Graphical representation of UV-Vis spectra for [6]MCPP and [6]MCPP@gas complexes.


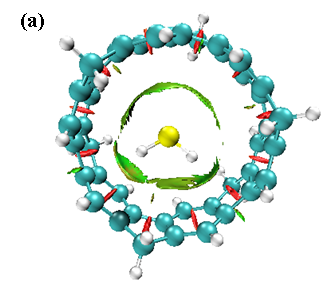

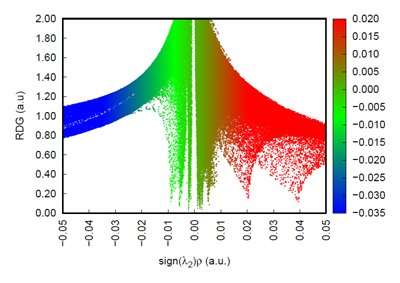


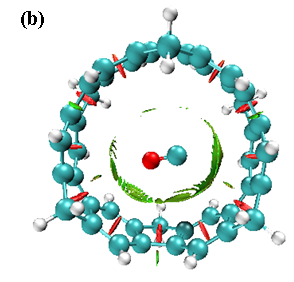

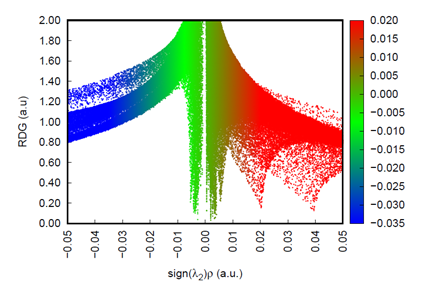


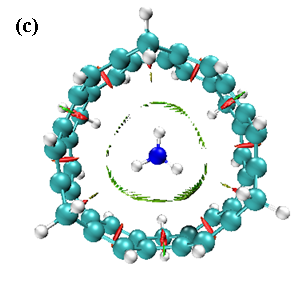

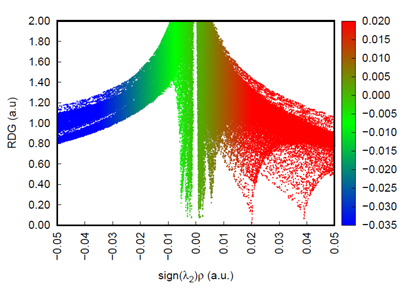


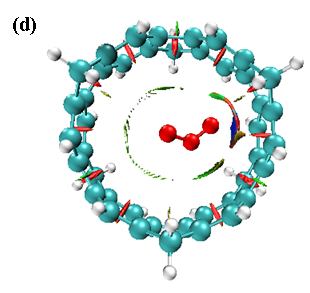

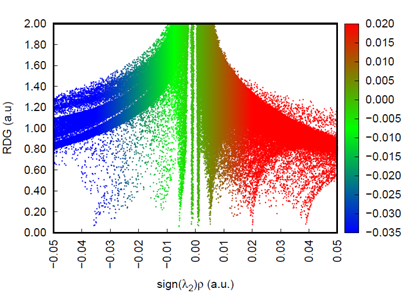


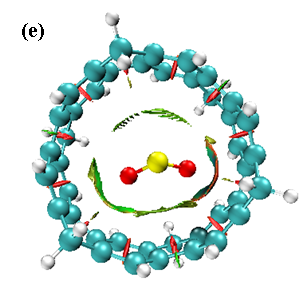

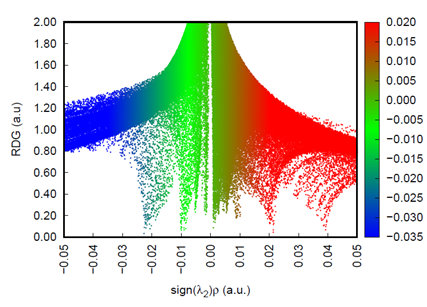


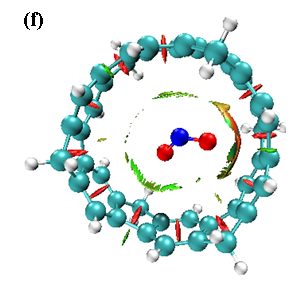

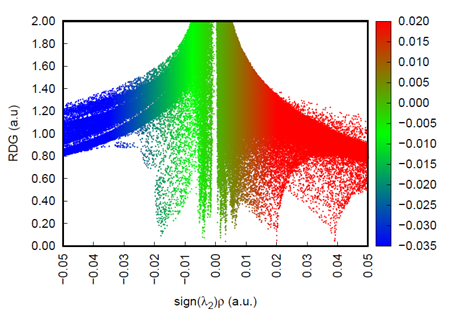

**Figure S3**. Reduced density gradient (RDG) graph (right side) and 3D isosurface map (left side) for (a) [6]MCPP@H₂S, (b) [6]MCPP@CO, (c) [6]MCPP@NH₃, (d) [6]MCPP@O₃, (e) [6]MCPP@SO₂, and (f) [6]MCPP@NO₂.


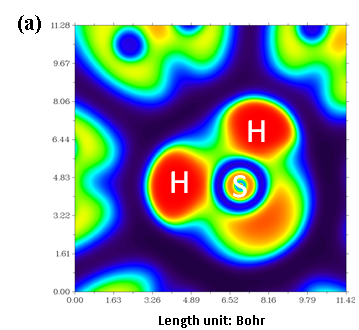

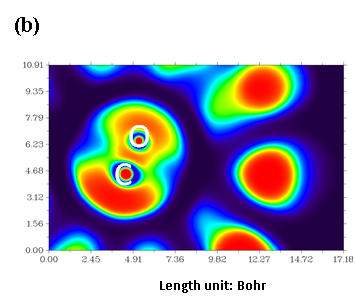


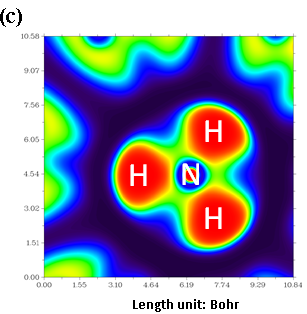

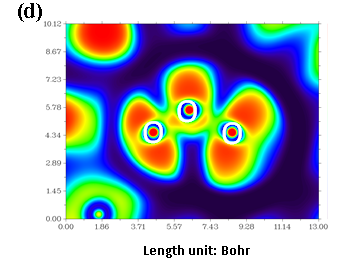


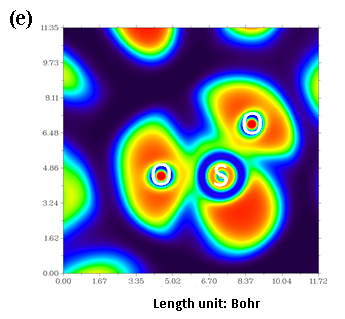

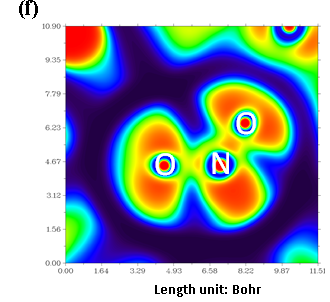


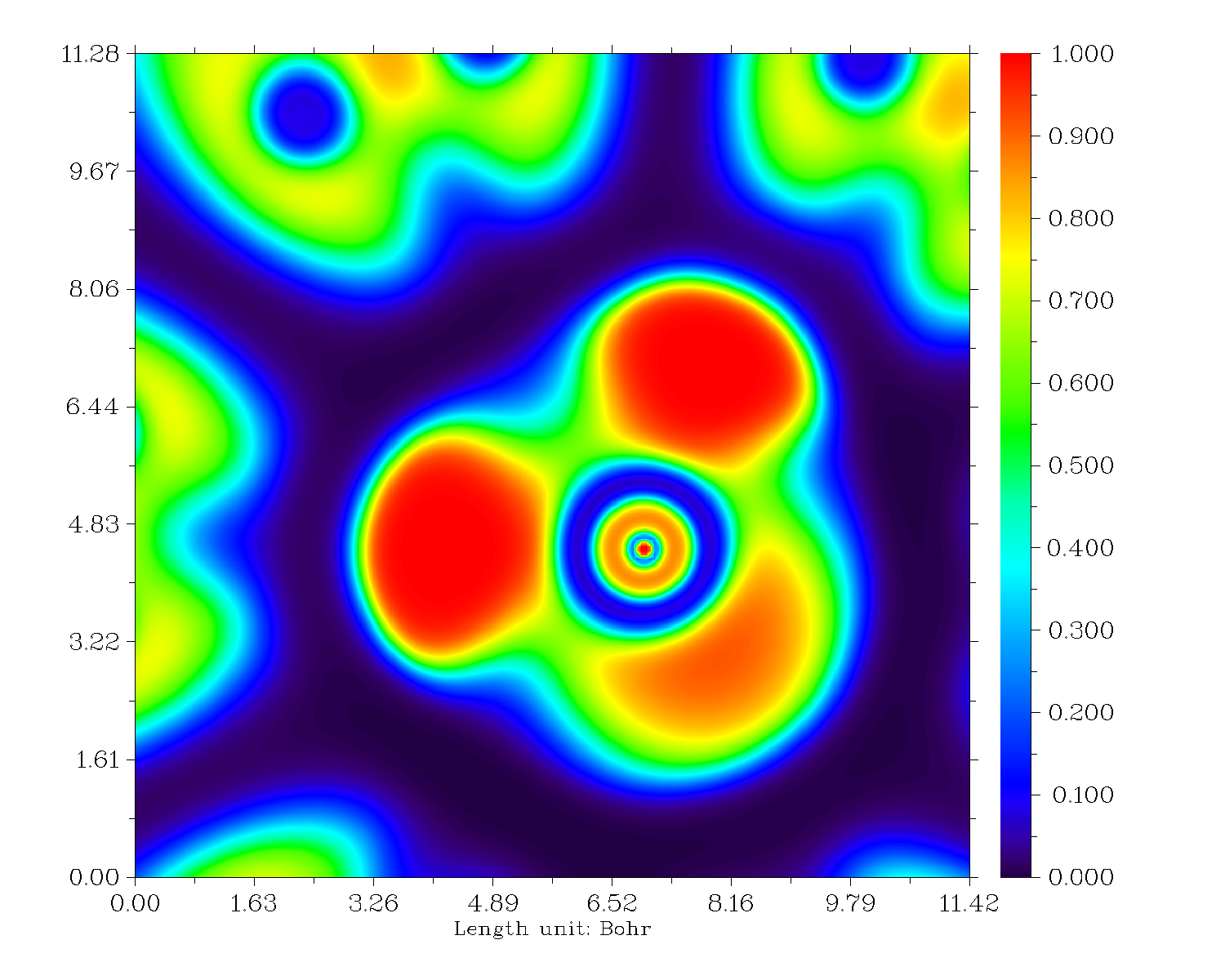


**Figure S4.** Electron Localization Function (ELF) maps of (a) [6]MCPP@H₂S, (b) [6]MCPP@CO, (c) [6]MCPP@NH₃, (d) [6]MCPP@O₃, (e) [6]MCPP@SO₂, and (f) [6]MCPP@NO₂.

**Cartesian coordinates (Angstrom):**

**[6]MCPP**

**X Y Z**

C -1.22609100 3.84516700 -1.05225600

C -0.79157700 4.00427100 0.28860500

C -1.63077400 3.62971800 1.34915300

C -2.78826200 2.91873800 1.05219000

C -3.13624100 2.61364100 -0.28869600

C -2.40504200 3.17037400 -1.34923400

C -3.56460500 2.00138200 2.00325200

C -3.94402300 0.86106100 1.05220300

C -3.94866300 -0.49733200 1.34920800

C -3.83190600 -1.40892200 0.28866700

C -3.92234100 -0.95508000 -1.05219900

C -3.95967600 0.40279600 -1.34919100

C -3.86465000 1.31694300 -0.28866400

C -3.07289300 -2.68795000 0.28861700

C -2.71763300 -2.98448900 -1.05225400

C -3.51563200 -2.08590500 -2.00329600

C -2.32866100 -3.22716400 1.34915400

C -1.13387600 -3.87349100 1.05219800

C -0.69558200 -4.02203500 -0.28866300

C -1.54343900 -3.66753800 -1.34922900

C 0.04881600 -4.08667200 2.00329900

C 1.22609100 -3.84516900 1.05225500

C 0.79157800 -4.00427100 -0.28860500

C 2.40504300 -3.17037500 1.34923300

C 3.13624000 -2.61364100 0.28869600

C 2.78826100 -2.91873600 -1.05219000

C 1.63077400 -3.62971700 -1.34915300

C 3.86464900 -1.31694300 0.28866500

C 3.94402400 -0.86106100 -1.05220200

C 3.56460600 -2.00138200 -2.00325200

C 3.95967500 -0.40279600 1.34919100

C 3.92234000 0.95508000 1.05219900

C 3.83190700 1.40892300 -0.28866700

C 3.94866400 0.49733200 -1.34920700

C 3.51563100 2.08590600 2.00329700

C 2.71763300 2.98448900 1.05225400

C 3.07289300 2.68795000 -0.28861700

C 1.54343900 3.66753900 1.34922900

C 0.69558200 4.02203400 0.28866300

C 1.13387600 3.87348900 -1.05219800

C 2.32866100 3.22716300 -1.34915400

C -0.04881600 4.08667100 -2.00329900

H -1.29246200 3.71117800 2.37672200

H -2.63878500 2.91258100 -2.37680400

H -4.45783600 2.50312500 2.40014400

H -2.94973800 1.65598800 2.83870800

H -3.84208100 -0.82857700 2.37678200

H -3.86115200 0.73649600 -2.37677800

H -4.39654900 -2.60880000 -2.40040600

H -2.90905500 -1.72578900 -2.83859800

H -2.56853300 -2.97510600 2.37673300

H -1.20328100 -3.74088000 -2.37680300

H 0.06108900 -5.11101900 2.40040300

H 0.04034000 -3.38130500 2.83860200

H 2.63878400 -2.91258200 2.37680400

H 1.29246100 -3.71117700 -2.37672200

H 4.45783700 -2.50312500 -2.40014200

H 2.94974000 -1.65598800 -2.83870800

H 3.86115000 -0.73649500 2.37677900

H 3.84208300 0.82857700 -2.37678100

H 4.39654800 2.60880000 2.40040600

H 2.90905400 1.72579000 2.83859800

H 1.20328100 3.74088100 2.37680300

H 2.56853300 2.97510500 -2.37673300

H -0.06108900 5.11101800 -2.40040300

H -0.04034000 3.38130400 -2.83860300

**[6]MCPP@H_2_S**

**X Y Z**

C -3.81707100 1.28533700 1.06404500

C -3.98142600 0.85402200 -0.27985400

C -3.60132900 1.69164000 -1.34376000

C -2.86065200 2.83457000 -1.04623600

C -2.54715200 3.17400500 0.29846600

C -3.12669000 2.45916900 1.36223700

C -1.92992800 3.58814300 -1.99085600

C -0.78857300 3.94939400 -1.04536700

C 0.57319300 3.93716800 -1.34350800

C 1.48055200 3.79118400 -0.27983900

C 1.03094800 3.89318900 1.06465500

C -0.32814100 3.96469400 1.36254500

C -1.24372800 3.87762200 0.29842100

C 2.74400800 3.02063300 -0.28041500

C 3.03720200 2.66473600 1.06291100

C 2.15018000 3.46747800 2.00904600

C 3.28060300 2.27608500 -1.34509400

C 3.90189700 1.06533300 -1.04794300

C 4.04016900 0.62541800 0.29570900

C 3.71241200 1.48264000 1.36003900

C 4.08751400 -0.11603700 -1.99390900

C 3.83454200 -1.28494800 -1.04804000

C 3.99771900 -0.85381200 0.29567700

C 3.14466800 -2.45796700 -1.34524700

C 2.56694100 -3.17114000 -0.28060100

C 2.88010100 -2.83276400 1.06273000

C 3.62147600 -1.69102600 1.35997700

C 1.26204400 -3.86911400 -0.27996500

C 0.80754900 -3.94570100 1.06460100

C 1.94940800 -3.58446800 2.00887300

C 0.34789800 -3.96337700 -1.34359600

C -1.01232300 -3.89837100 -1.04533800

C -1.46259400 -3.80083900 0.29847400

C -0.55338300 -3.93988200 1.36258800

C -2.13127500 -3.47316300 -1.99082200

C -3.01725300 -2.66748200 -1.04624300

C -2.72388600 -3.02426600 0.29849000

C -3.69142600 -1.48412700 -1.34378000

C -4.02334000 -0.62630700 -0.27989000

C -3.88416800 -1.06629500 1.06405000

C -3.26195300 -2.27760800 1.36224100

C -4.07426000 0.11590200 2.00907600

H -3.69001700 1.35469400 -2.37237200

H -2.86454100 2.68915300 2.39068200

H -2.41295000 4.48379200 -2.40847100

H -1.59810200 2.97149900 -2.83241600

H 0.90320700 3.82319700 -2.37175500

H -0.66463300 3.87370100 2.39121900

H 2.67997500 4.33490200 2.42995300

H 1.78343000 2.86765100 2.84819400

H 3.02886900 2.51937700 -2.37319300

H 3.77889400 1.13886100 2.38803100

H 5.10196000 -0.14508400 -2.41869600

H 3.38068100 -0.09575700 -2.82947200

H 2.87945400 -2.68635100 -2.37336700

H 3.70820800 -1.35209300 2.38809100

H 2.42946000 -4.48077700 2.42898100

H 1.61764400 -2.96542300 2.84861800

H 0.68383600 -3.86836700 -2.37185700

H -0.88410300 -3.83011800 2.39130600

H -2.66446900 -4.33995300 -2.40832500

H -1.76480000 -2.87656700 -2.83245100

H -3.76049300 -1.14260900 -2.37238900

H -3.01338900 -2.52218100 2.39070300

H -5.09208400 0.14490500 2.42501700

H -3.37609700 0.09602800 2.85216800

S -0.03674200 0.00137900 -0.13937000

H -0.99634200 -0.98655000 -0.07862900

H -0.94857900 1.03398500 -0.08692500

**[6]MCPP@CO**

**X Y Z**

C 0.41724000 -3.98399200 -1.05610600

C -0.03844500 -4.04435800 0.28803500

C 0.86525400 -3.87307900 1.35143200

C 2.14962700 -3.42238000 1.05273200

C 2.55137000 -3.19595800 -0.29125900

C 1.71990000 -3.58855800 -1.35491000

C 3.10574400 -2.70230800 1.99813900

C 3.72285800 -1.67437400 1.05503100

C 4.02774800 -0.34806500 1.35559700

C 4.09440600 0.57079900 0.29340000

C 4.09329600 0.11176400 -1.05119100

C 3.84725700 -1.22672000 -1.35165600

C 3.54305400 -2.09650400 -0.28959900

C 3.61585200 1.97207300 0.29476000

C 3.33462100 2.33756500 -1.04913000

C 3.93290300 1.30020400 -1.99423200

C 3.00227800 2.65706000 1.35840400

C 1.95415500 3.52564200 1.05944100

C 1.55625600 3.75622200 -0.28477400

C 2.32505400 3.25066700 -1.34783800

C 0.84221500 3.97144700 2.00349400

C -0.35392800 3.98231800 1.05747000

C 0.10402600 4.04205000 -0.28622100

C -1.65610100 3.58458600 1.35477200

C -2.48652700 3.19172500 0.29014300

C -2.08164100 3.41310500 -1.05327500

C -0.79736300 3.86563800 -1.35044800

C -3.48058100 2.09502400 0.29041500

C -3.66043400 1.66896900 -1.05251000

C -3.03367300 2.68785200 -1.99863300

C -3.78155500 1.22723500 1.35509500

C -4.03142500 -0.11191200 1.05839500

C -4.03826000 -0.57378400 -0.28510000

C -3.96906400 0.34256400 -1.34899100

C -3.86047800 -1.29604400 2.00432600

C -3.26625200 -2.33432800 1.05827300

C -3.55543600 -1.97385900 -0.28524200

C -2.25305700 -3.24452000 1.35493900

C -1.48942700 -3.75381100 0.28988700

C -1.89098200 -3.52361000 -1.05311400

C -2.94165200 -2.65727700 -1.34957000

C -0.78087600 -3.96790300 -1.99987100

H 0.51814500 -3.88416900 2.38052600

H 2.00484400 -3.38780300 -2.38358300

H 3.86381400 -3.38538400 2.40915600

H 2.58925700 -2.23879800 2.84498500

H 3.99546500 -0.00300500 2.38497400

H 3.68152900 -1.53089900 -2.38109000

H 4.89969400 1.63019400 -2.40240700

H 3.27796100 1.07783000 -2.84292500

H 3.18691500 2.36166100 2.38716700

H 2.00900600 3.39443900 -2.37697000

H 1.03906800 4.96923700 2.42288500

H 0.70520200 3.28415700 2.84476000

H -1.94280600 3.38579500 2.38327300

H -0.44794200 3.87293900 -2.37874100

H -3.78608000 3.36924600 -2.42251700

H -2.51092500 2.21352000 -2.83536900

H -3.61006700 1.53218400 2.38316800

H -3.93246500 -0.00574100 -2.37690500

H -4.82205600 -1.62762000 2.42296800

H -3.19648900 -1.06713400 2.84390800

H -1.93207500 -3.38435600 2.38296300

H -3.12802400 -2.36140800 -2.37771300

H -0.97996000 -4.96370800 -2.42294400

H -0.64471900 -3.27715900 -2.83839400

C -0.62425900 -0.01014800 0.61767600

O -0.65493300 0.03998400 -0.53753900

**[6]MCPP@NH_3_**

**X Y Z**

C -1.00623500 -3.86405100 -1.01311300

C -1.45689700 -3.77184900 0.33105200

C -0.54872100 -3.92155200 1.39519800

C 0.81282200 -3.93252600 1.09803100

C 1.26799400 -3.85668300 -0.24617800

C 0.35389300 -3.93819400 -1.31069000

C 1.95864800 -3.57907000 2.04200200

C 2.88885300 -2.82307300 1.09726800

C 3.63132300 -1.68105100 1.39332500

C 4.00921300 -0.84448900 0.32772200

C 3.84604200 -1.27836900 -1.01545600

C 3.15426300 -2.45096900 -1.31180200

C 2.57445400 -3.16125300 -0.24626100

C 4.05102300 0.63587900 0.32363700

C 3.91049400 1.07097300 -1.02191800

C 4.10251100 -0.11250500 -1.96398100

C 3.72422100 1.49899000 1.38473700

C 3.04753700 2.67988200 1.08344100

C 2.74930900 3.02807100 -0.26116700

C 3.28583600 2.27959200 -1.32367900

C 2.16447200 3.49243900 2.02607500

C 1.03879200 3.90597600 1.08249800

C 1.48523600 3.79787900 -0.26190200

C -0.32061300 3.97210900 1.38331200

C -1.23789200 3.86997900 0.32221800

C -0.78605000 3.93595500 -1.02339700

C 0.57453600 3.92965100 -1.32482300

C -2.54362400 3.17198500 0.32873000

C -2.86094800 2.82971900 -1.01314200

C -1.92686700 3.56768700 -1.96517700

C -3.12601800 2.46578800 1.39692700

C -3.82487900 1.29566700 1.10482900

C -3.99467800 0.86261500 -0.23786700

C -3.61112700 1.69239800 -1.30588200

C -4.08822900 0.12751800 2.05153000

C -3.89514400 -1.05657900 1.10780900

C -4.04037600 -0.61728500 -0.23617900

C -3.26508500 -2.26428400 1.40170600

C -2.72261900 -3.00365100 0.33417900

C -3.01819600 -2.64550800 -1.00796300

C -3.70630300 -1.46956800 -1.30213000

C -2.11277600 -3.41663500 -1.96031900

H -0.88025300 -3.81742800 2.42430300

H 0.68981600 -3.83815800 -2.33831300

H 2.43984700 -4.47893100 2.45291000

H 1.63195000 -2.96765400 2.88956700

H 3.72321900 -1.34344600 2.42162600

H 2.89051800 -2.68119400 -2.33973400

H 5.12021600 -0.14122400 -2.38053500

H 3.40301100 -0.09637900 -2.80595500

H 3.79923700 1.16287900 2.41489400

H 3.03670600 2.52066500 -2.35294400

H 2.69719600 4.36527600 2.43167000

H 1.80524900 2.90470800 2.87722900

H -0.65455400 3.88975300 2.41362700

H 0.90277000 3.81419600 -2.35363000

H -2.40401500 4.45943900 -2.39774700

H -1.59549000 2.93524200 -2.79508800

H -2.86173800 2.69948700 2.42424100

H -3.70017300 1.35036200 -2.33246700

H -5.10821500 0.15876600 2.46228500

H -3.39508700 0.10791600 2.89885600

H -3.01480500 -2.51126400 2.42942000

H -3.76981600 -1.12293600 -2.32884400

H -2.62928300 -4.26933400 -2.42462200

H -1.72901600 -2.77696700 -2.76131500

N -0.37087200 -0.29227000 -1.16691500

H -1.30291500 -0.10746700 -0.82367800

H 0.00390500 -1.18187300 -0.86704300

H 0.26864400 0.48181100 -1.04951300

**[6]MCPP@O_3_**

**X Y Z**

C -2.09200500 -3.40229300 1.11513300

C -1.70735500 -3.64845500 -0.23252500

C -2.44522000 -3.09368000 -1.29619700

C -3.39822400 -2.12698000 -0.99471300

C -3.65450400 -1.74842000 0.35250700

C -3.08039500 -2.47299000 1.41645900

C -3.93030600 -1.05270300 -1.93542100

C -4.04111900 0.13202300 -0.98366500

C -3.69286000 1.44534100 -1.27850900

C -3.31994300 2.28561800 -0.21099900

C -3.52711600 1.87216600 1.13746700

C -3.92450100 0.57471200 1.43071800

C -4.05861000 -0.33233700 0.35849100

C -2.29036500 3.33033000 -0.21452800

C -1.87252300 3.54202300 1.13053600

C -2.85396300 2.86359900 2.07991500

C -1.45409900 3.69645800 -1.28632300

C -0.13959300 4.04336000 -0.99692000

C 0.32968700 4.07287000 0.34479100

C -0.57288300 3.94123300 1.41874100

C 1.04379800 3.95112700 -1.95201100

C 2.12953800 3.43761500 -1.01416100

C 1.75355700 3.69341700 0.33494000

C 3.12075800 2.51043000 -1.31807900

C 3.69337400 1.78684600 -0.25581700

C 3.44338000 2.16877900 1.09222500

C 2.49701800 3.14240700 1.39597400

C 4.08363000 0.36608000 -0.25992900

C 4.06702900 -0.09743300 1.08602900

C 3.98210100 1.09545300 2.03242900

C 3.96589400 -0.54363500 -1.32650100

C 3.59492000 -1.85089500 -1.02870400

C 3.40174800 -2.26801800 0.31768300

C 3.75995600 -1.42109500 1.38285100

C 2.91300800 -2.83593900 -1.97131800

C 1.92627500 -3.50216400 -1.02046700

C 2.35007500 -3.29856600 0.32466700

C 0.62348500 -3.88505000 -1.31088300

C -0.28066100 -4.00083000 -0.23868700

C 0.18843400 -3.96717600 1.10589700

C 1.51084000 -3.65276700 1.39610300

C -1.00077700 -3.89975800 2.05595500

H -2.13329500 -3.24780800 -2.32457400

H -3.24597500 -2.16804200 2.44534300

H -4.90253300 -1.32990300 -2.36863800

H -3.24195100 -0.85005700 -2.76170000

H -3.50115700 1.74025500 -2.30483200

H -3.91306100 0.21951200 2.45680400

H -3.57478800 3.57890700 2.50296900

H -2.35141400 2.36901900 2.91719500

H -1.75218800 3.49964400 -2.31102000

H -0.21510300 3.93430400 2.44401400

H 1.30362100 4.92894400 -2.38369900

H 0.85127500 3.26231400 -2.78036300

H 3.28155600 2.20328200 -2.34706400

H 2.19676300 3.30769600 2.42635200

H 4.96807300 1.36535800 2.43881700

H 3.31596500 0.90969700 2.88087700

H 3.94459700 -0.19238100 -2.35374400

H 3.58274000 -1.72102200 2.41126500

H 3.62538600 -3.55828000 -2.39651500

H 2.41196300 -2.33515200 -2.80561800

H 0.26794500 -3.87721200 -2.33661900

H 1.81566100 -3.47089600 2.42214400

H -1.24174500 -4.88568300 2.48035600

H -0.82778300 -3.21386700 2.89105800

O 0.19736400 -0.93140000 0.21923500

O -0.85566500 0.71709900 -1.01586200

O -0.20588100 -0.48230600 -0.99608900

**[6]MCPP@SO_2_**

**X Y Z**

C 1.13624000 -3.80691500 -0.94923200

C 0.71050200 -3.95968300 0.39824100

C 1.57477500 -3.62828900 1.46145400

C 2.74432600 -2.94158900 1.15932500

C 3.08777700 -2.64002900 -0.19010500

C 2.33655800 -3.17426500 -1.25254800

C 3.56782300 -2.06621100 2.09835000

C 3.99264700 -0.94949100 1.14968000

C 4.05982100 0.40895400 1.44047400

C 3.94922400 1.31728200 0.36985200

C 4.02841600 0.85879800 -0.97285000

C 4.02190200 -0.50033500 -1.26431000

C 3.87814200 -1.40137800 -0.19435800

C 3.19803000 2.58780100 0.36826400

C 2.83118400 2.87764900 -0.97568700

C 3.61102500 1.97463600 -1.92279000

C 2.48183500 3.15792300 1.43765800

C 1.28659000 3.80986000 1.14860600

C 0.83251700 3.94724300 -0.19289000

C 1.66438300 3.57882700 -1.26588300

C 0.12764500 4.07112000 2.10516700

C -1.06511900 3.85735200 1.17869500

C -0.64314000 3.98255100 -0.17388000

C -2.26751900 3.23183300 1.49880500

C -3.02453900 2.68030700 0.44857300

C -2.68516300 2.96026100 -0.90598300

C -1.51097700 3.63864400 -1.22494400

C -3.81821200 1.43586200 0.46879200

C -3.95482600 0.98461200 -0.87204100

C -3.51077400 2.07947100 -1.83451700

C -3.94005900 0.52974900 1.54126500

C -3.94627900 -0.83074900 1.24665800

C -3.88681500 -1.28282900 -0.10133000

C -4.02413200 -0.37534300 -1.16585200

C -3.53610800 -1.96640200 2.18049700

C -2.75235000 -2.85670800 1.22062800

C -3.11889900 -2.54173900 -0.11916300

C -1.59557000 -3.57939600 1.49603000

C -0.76425700 -3.93302900 0.41510300

C -1.21338200 -3.75886600 -0.92372900

C -2.39855700 -3.08442100 -1.20020100

C -0.05283800 -3.97008000 -1.88743300

H 1.24201600 -3.71324200 2.49181200

H 2.56588500 -2.90738600 -2.27895300

H 4.43326200 -2.60781500 2.50797500

H 2.98188900 -1.69551600 2.94558800

H 3.96886000 0.75087400 2.46737100

H 3.89034500 -0.83461400 -2.28838800

H 4.47713600 2.49286100 -2.36009900

H 2.99386100 1.60495500 -2.74744300

H 2.73853000 2.91938900 2.46555100

H 1.31289500 3.65180400 -2.29068100

H 0.15370500 5.09547600 2.50530300

H 0.12455500 3.38682900 2.95991100

H -2.50357900 3.00002300 2.53327300

H -1.18254700 3.69724400 -2.25807500

H -4.36294400 2.62512600 -2.26497600

H -2.91283400 1.67289500 -2.65513900

H -3.80935300 0.86457400 2.56621200

H -3.92669800 -0.70861800 -2.19290700

H -4.41065600 -2.48864100 2.59554200

H -2.93024500 -1.62107500 3.02447500

H -1.24471900 -3.67849100 2.51897400

H -2.63680500 -2.80144200 -2.22000500

H -0.08086100 -4.96548000 -2.35426400

H -0.04884900 -3.22476800 -2.68951300

S -0.55485700 -0.02001600 -0.72849600

O -1.05747100 -0.10109700 -2.27891900

O 1.00419100 -0.46548200 -0.56857400

**[6]MCPP@NO_2_**

**X Y Z**

C -2.52142900 3.09065100 -0.94803600

C -2.15257100 3.39468500 0.39000800

C -2.79998800 2.75966300 1.46774000

C -3.62804500 1.67711500 1.18675600

C -3.86014600 1.26459300 -0.15476600

C -3.39785400 2.04605700 -1.22944600

C -4.00468200 0.54376000 2.13673300

C -3.94683200 -0.65152800 1.18969800

C -3.42287300 -1.90883000 1.47549100

C -2.95224700 -2.68568600 0.40068500

C -3.22714000 -2.29549600 -0.94222200

C -3.82130200 -1.07060300 -1.22627500

C -4.06559800 -0.19672400 -0.15290000

C -1.78340100 -3.58284600 0.38189600

C -1.35650200 -3.71654500 -0.96754900

C -2.42169200 -3.15991000 -1.90339600

C -0.88625100 -3.82680800 1.43886400

C 0.46275800 -3.96865100 1.13015700

C 0.91249600 -3.92993500 -0.21955000

C -0.01418300 -3.91724000 -1.27723900

C 1.64556300 -3.74046000 2.06578300

C 2.64382600 -3.08726100 1.11523100

C 2.28244900 -3.38376100 -0.22798700

C 3.51101100 -2.03762600 1.40776300

C 3.97152200 -1.24529900 0.33987700

C 3.75565900 -1.65822100 -1.00454700

C 2.93545500 -2.74388100 -1.29744400

C 4.17210300 0.21858000 0.33690000

C 4.07268800 0.66893700 -1.00851300

C 4.13983500 -0.52702300 -1.95220700

C 3.93784200 1.10864100 1.40171100

C 3.37711300 2.34815900 1.10493700

C 3.10397100 2.72366100 -0.23929500

C 3.56580100 1.93111300 -1.30577400

C 2.58203600 3.24104100 2.05259600

C 1.49404300 3.75638100 1.11575800

C 1.91996500 3.60600800 -0.23305900

C 0.15084000 3.95203800 1.42588300

C -0.77927500 3.93464500 0.37039000

C -0.33209100 3.94873100 -0.98055200

C 1.01881700 3.81893200 -1.29080100

C -1.51116600 3.69936700 -1.91273300

H -2.49634000 2.96111000 2.49079700

H -3.53137600 1.71180600 -2.25332200

H -5.01006700 0.68303100 2.56070200

H -3.30436100 0.44832000 2.97235700

H -3.18461300 -2.17979000 2.49962400

H -3.86546300 -0.71585200 -2.25060300

H -3.03246500 -3.95526300 -2.35474400

H -1.98699200 -2.56614600 -2.71289600

H -1.19745600 -3.69079300 2.47034800

H 0.32191800 -3.84926000 -2.30752300

H 2.03146700 -4.68610000 2.47444800

H 1.39068400 -3.09656900 2.91367300

H 3.64700900 -1.71430400 2.43556700

H 2.64365200 -2.94441300 -2.32403700

H 5.15013100 -0.66559800 -2.36461700

H 3.44975200 -0.43447200 -2.79687400

H 3.98375700 0.76462700 2.43080800

H 3.33471800 2.19625400 -2.33312900

H 3.19752500 4.06003500 2.45327300

H 2.17417100 2.68978600 2.90597300

H -0.18261400 3.90735800 2.45844300

H 1.32786500 3.66847700 -2.32078400

H -1.88084300 4.63120900 -2.36523700

H -1.25731400 3.01227700 -2.72647900

N -0.60458500 0.20358700 -0.94507400

O -0.91201300 -0.09642400 -2.11986900

O -0.85221200 -0.41000000 0.10691400
